# Laminin 332 expression levels predict clinical outcomes and chemotherapy response in patients with pancreatic adenocarcinoma

**DOI:** 10.3389/fcell.2023.1242706

**Published:** 2023-09-15

**Authors:** Bilge Sari, Ozcan Gulbey, Kevin J. Hamill

**Affiliations:** ^**1**^ Institute of Life Course and Medical Sciences, University of Liverpool, Liverpool, United Kingdom; ^**2**^ Translational and Clinical Research Institute, Newcastle University, Newcastle upon Tyne, United Kingdom

**Keywords:** pancreatic adenocarcinoma, laminin family, survival outcomes, bioinformatics analysis, drug sensitivity

## Abstract

Poor outcomes and chemotherapy resistance for patients with pancreatic adenocarcinoma (PAAD) are a challenge worldwide, and new or improved prognostic biomarkers are urgently required. Individual laminin family members have been established as cancer-associated markers, predicting patient outcomes in many cancer types, including PAAD. Here, we used multiple modalities including RNAseq and gene chip, and genomic and proteomic data to examine the relationships of all laminin genes in PAAD with clinical outcomes. These analyses identified that LAMA3, LAMB3, and LAMC2 expression levels are increased at the mRNA and protein levels in PAAD tumours with evidence of co-regulation. Increased expression of all three genes was associated with decreased promoter methylation status, TP53 mutations, and altered receptor tyrosine kinase (RTK) pathways. Clinically, high LAMA3, LAMB3, and LAMC2 transcript abundance was each related to an advanced histological grade. Moreover, high expression of these genes individually predicted poor patient survival, while a signature of combined high expression of LAMA3, LAMB3, and LAMC2 was a stronger predictor of patient outcomes than each gene alone. Interestingly, cell lines with high expression of LM332 chains were not sensitive to the commonly used PAAD chemotherapy drugs paclitaxel and gemcitabine; however, increased sensitivity was evident for erlotinib, afatinib, gefitinib, and cetuximab epidermal growth factor (EGFR) RTK inhibitors. To explore possible mechanisms, we investigated co-expressed genes, identifying eight hub genes, namely, *GJB3*, *ITGB6*, *SERPINB5*, *GPRC5A*, *PLEK2*, *TMPRSS4*, *P2RY2*, and *TRIM29*, which are co-expressed with all three of LAMA3, LAMB3, and LAMC2. Of these, only SERPINB5 provided a stronger predictive value than the laminin-encoding genes. Together, these multiple integrated analyses suggest that the combined expression of LM332 is a useful prognostic biomarker for PAAD and could help patient stratification and therapeutic selection.

## Introduction

Pancreatic adenocarcinoma (PAAD) ranks either fourth or fifth among cancer-related deaths in developed countries (Arkin et al., 2001), with an estimated 60,430 new cases and 48,220 deaths in the United States in 2022 (Siegel et al., 2021). PAAD has such high mortality rates due to the difficulty of early detection of the disease; approximately 85% of cases are not eligible for surgical resection upon discovery (Pereira et al., 2020). Moreover, although combined therapy models of chemotherapy, immunotherapy, and radiotherapy have been used to treat patients, chemotherapy resistance continues to negatively affect therapeutic outcomes (Von Hoff et al., 2013). Thus, survival times have not improved substantially, and the 5-year survival rate remains less than 10% (Mizrahi et al., 2020). Identifying effective molecular biomarkers whose change in expression can help stratify patients could provide valuable insights for treatment decisions. If these prognostic biomarkers also provided an indication of resistance or sensitivity to specific compounds, they could open possibilities toward personalised medicine development.

The laminins are a family of large extracellular glycoproteins that are core components of the basement membrane (BM). Within BMs, the laminins form independent structural networks, which then interact with the other structural components of the BM, the collagen IV network, via linker proteins including nidogen, agrin, and perlecan (Groffen et al., 1998; Noonanssli et al., 1991; Yap et al., 2019). Cells bind laminins via cell surface receptors including integrins and dystroglycan (Timpl et al., 2000). There are 11 laminin-encoding genes in humans, broken into five α-chain encoding genes (LAMA1–5), three β-chain encoding genes (LAMB1–3), and three γ-chain encoding genes (LAMC1–3). A fourth LAMB gene, *LAMB4*, has been identified, which appears to be expressed at very low levels and is likely a pseudogene (Aumailley, 2013). Each laminin protein is an obligate heterotrimer, comprising one α, one β, and one γ chain, and these trimers are named based on their chain composition, e.g., laminin α3β3γ2 is known as LM332 (Hamill et al., 2009). When considering laminins at the functional level, it is therefore important to consider not only single chains but rather family-wide effects. Each laminin has a specific expression profile and plays distinct roles in the homoeostasis of different tissues, owing to their multiple functional roles including regulating cell adhesion and proliferation, differentiation, and migration (Aumailley, 2013). These essential proteins are also directly associated with cancer progression through the regulation of metastasis, invasion, and cancer-associated signalling pathway activation during cancer development (Chang et al., 2019; Chavda et al., 2022).

Numerous studies have demonstrated the prognostic value of laminins and survival in a range of cancers. For example, in colorectal cancer, an increased LAMA4 to LAMA5 ratio was related to increased BM permeability and poor survival in patients (V Galatenko et al., 2018); in ovarian cancer, LAMC1 and the LAMA1 to LAMA5 ratio were negatively associated with tumour immune infiltrates (TILs), whereas LAMA4 and LAMB1 predicted tumour purity, and LAMB3 and LAMC2 correlated with platinum resistance (Diao and Yang, 2021). Higher levels of LM511 have been connected with cell migration or metastasis or cell malignancy in gliomas, melanomas, prostate, and breast cancers (Chia et al., 2007; Brar et al., 2003; Kawataki et al., 2007; Oikawa et al., 2010).

In pancreatic cancer, numerous laminin studies have independently connected expression levels of single laminin genes with outcomes, although most have focused on pancreatic ductal adenocarcinoma (PDAC). Specifically, mRNA expression levels of the laminin family in PDAC blood cells suggested that LAMA3 and LAMC2 had the highest prognostic values (Yang et al., 2019). Independently, a strong correlation between LAMA3 mRNA and AC245041.2 lncRNA has been demonstrated with high expression of both associated with worse survival and with KRAS mutations (Tian et al., 2021). Functional enrichment analyses identified five hub genes including LAMA3, which might be relevant to poor survival PAAD and possible drug sensitivity to the RTK inhibitor dasatinib in pancreatic cancer cell lines (Wei et al., 2019). High expression of LAMB3 has also, independently, been detected in PDAC, and the knockdown of LAMB3 reduced proliferation, invasion, and metastasis likely by affecting the PI3K/AKT signalling pathway (Zhang et al., 2019). High expression of LAMC2 has been detected in pancreatic tumours, which modulated cancer microenvironment acidity (Wang et al., 2020; Erice et al., 2023; Okada et al., 2021). Analysis of the proteome of PDAC tissues with that of adjacent normal counterparts described LMγ2 (the protein product of LAMC2) as a strong prognostic candidate (Kosanam et al., 2013). Functionally, upregulated LMγ2 has been shown to mediate tumourigenesis, metastasis, and epithelial–mesenchymal transition (EMT) by regulating EGFR/ERK1/2/AKT/mTOR cascades in PDAC (Erice et al., 2023; Kirtonia et al., 2023). High LAMC2 has also been suggested as promoting gemcitabine chemotherapy resistance by activating EMT in PDAC (Okada et al., 2021). Cancer-associated fibroblasts have been shown to induce LM332 production and had a positive correlation with integrins in promoting cell differentiation and cancer cell invasion in PDAC cells (Cavaco et al., 2019).

The prior laminin studies have each highlighted the value of analysing laminins in PDAC. With the wealth of large data sets now available, we sought to build on these studies by comprehensively analysing the association of the laminin family with patient features, the drivers underlying the laminin expression dysregulation, and mechanisms mediating poor outcomes in PAAD across multiple modalities and at different levels including mutational statues, gene regulation, protein, and transcript abundance. Herein, we asked whether mutation status, clinicopathological background, or altered regulatory mechanisms in patients with PAAD are associated with changed laminin expression, we analysed the association between survival and mRNA expression of individual laminins, laminin combinations, and co-expressed genes, and identified predictors of therapeutic response based on laminin expression. Together, this integrated large cohort study interrogation identified a laminin expression signature that predicts patient outcomes and therapeutic response, which could now become a useful tool in patient stratification.

## Methods

### Expression profiling of laminin family

Differential expression of three laminin genes in PAAD was analysed using the TNM Plotter (https://tnmplot.com/analysis/) accessed on May 2023, described in detail in Bartha and Győrffy (2021). This tool enabled a comparison of gene chip data on paired tumour and adjacent tissues; and RNA-seq data on tumour tissues and normal tissues using data extracted from The Cancer Genome Atlas (TCGA) and genotype-tissue expression (GTEx) datasets. Data analysis features of the TNM-plotter pipeline were developed in R version 3.6.1. Direct comparisons of normal and cancer samples were made using the Mann–Whitney *U* test, and comparisons of normal and matched tissues with adjacent samples were made using the Wilcoxon test. *p*-values for each gene based were calculated using the Mann–Whitney *U* test results. Volcano plots showing –log10*p* values against Log_2_ fold changes to visualize differential expressions were generated (Bartha and Győrffy, 2021).

### Methylation and protein expression analysis

UALCAN (https://ualcan.path.uab.edu/) accessed on May 2023 was used to explore promotor methylation levels of the laminin gene family in normal and PAAD samples from the TCGA dataset generated using the Illumina Infinium HumanMethylation450 BeadChip, described in detail in Chandrashekar et al. (2017) and Chandrashekar et al. (2022). The reported beta (methylation) value corresponds to DNA methylation levels ranging from 0 (unmethylated) to 1 (fully methylated). A β-value ranging 0.7–0.5 indicates hyper-methylation, and a β-value ranging 0.3–0.25 indicates hypo-methylation. Welch’s *t*-test was used to analyse observed differences in expression levels between normal and cancer samples.

For protein expression data, PAAD data (normal *n* = 74, primary tumour *n* = 137) from the Clinical Proteomic Tumour Analysis Consortium (CPTAC) and the International Cancer Proteogenome Consortium (ICPC) were analysed using the UALCAN website. Data were generated from log_2_ spectral count ratio values from CPTAC normalised within each sample profile and then across samples. Data are plotted as z-values representing standard deviations from the median across the PAAD samples for each protein (Chandrashekar et al., 2017; Chandrashekar et al., 2022; Zhang et al., 2022; Chen et al., 2019).

### Mutation analysis

The open-source online tool cBioPortal (cBio Cancer Genomics Portal) (https://www.cbioportal.org/) was used to investigate mutation rates including the structural variant and putative copy-number alterations in laminin genes from Genomic Identification of Significant Targets in Cancer (GISTIC) data, methods described in detail in Cerami et al. (2012). Laminin genes were queried in five pancreatic carcinoma studies with 990 samples from 989 patients (accessed on May 2023). The correlation between these mutations and clinicopathological factors was evaluated by the chi-squared test. Laminin gene mutation and overall survival in PAAD were evaluated by the log-rank test.

### Survival and clinical characteristic analyses

Expression data for laminin transcripts in PAAD tumours in the TCGA cohort were extracted from https://xenabrowser.net/datapages/. Overall survival was defined as a time from diagnosis to death, with censoring at the date of last contact. The Kaplan–Meier method with the log-rank test was used to compare the overall survival (OS) rates between the low-expression (first tercile) and high-expression groups (third tercile). Univariate Cox regression was used to estimate the risk of death for high laminin gene expression. Chi-squared and Fisher’s exact tests were used to compare the high and low-expression groups for association with clinical features. *p*-values < 0.05 were considered statistically significant.

### Co-expressed genes and enrichment analyses

The LinkedOmics database was used to assess co-expressed genes with LM332 by querying individual laminin genes in the TCGA cancer cohort with 184 PAAD samples (https://www.linkedomics.org/) (accessed on May 2023), and correlation was evaluated by Pearson’s correlation. Overexpressed genes were entered into DAVID to determine functional annotations including biological process, molecular function, and cellular components. Biological pathways of key genes were presented by the KEGG pathway analysis of DAVID (https://david.ncifcrf.gov/). For the functional terms, genes in the collected datasets were analysed by Fisher’s exact test to calculate *p*-values. The Benjamini–Hochberg method (Hochberg and Benjamin, 1988) was used to calculate adjusted *p*-values for each term and to control the false discovery rate (FDR) (Huang et al., 2008; Sherman et al., 2022; Huang et al., 2009). The top 10 genes with positive and negative correlations with LM332 genes were examined in GeneCards (https://www.genecards.org/) (accessed on May 2023). The top 50 genes positively correlated for each laminin member were assessed for pathway and enrichment analyses using Metascape (https://metascape.org/), analysing the uploaded gene list with online sources including the GO biological process (BP), GO cellular component (CC), GO molecular function (MF) Reactome gene sets, and KEGG pathway. Genes were collected and grouped into clusters, and terms with *p* < 0.01 were accepted as statistically significant, and the Benjamini–Hochberg *p*-value correction was used. Connected regions in large protein–protein interaction networks were analysed using the clustering algorithm; “Molecular Complex Detection” (MCODE) (Bader and V Hogue, 2003) (accessed on May 2023) (Zhou et al., 2019).

### Immune infiltration analysis

TISIDB (http://cis.hku.hk/TISIDB/) (accessed on May 2023) integrating data about immune system interactions in different tumours from TCGA database was used to analyse the relation between immune molecules and LAMA3, LAMB3, and LAMC2 expression, described in detail in Ru et al. (2019). Differences were evaluated by Spearman’s test. The gene module of the TIMER2.0 online tool (accessed on May 2023) was used to analyse the LM gene correlation with immune infiltration. Purity-adjusted Spearman rhos were calculated for PAAD (Li et al., 2017).

### Drug sensitivity analysis

The Gene Set Cancer Analysis (GSCA) online tool (http://bioinfo.life.hust.edu.cn/GSCA/) was used to analyse the drug resistance/sensitivity correlation with the laminin gene expression and the genes positively correlated with laminins, described in detail in Liu et al. (2018) and Rees et al. (2016). This tool analyses the IC_50_ concentration of 265 small molecules in 860 cell lines and the corresponding mRNA expression from Genomics of Drug Sensitivity in Cancer (GDSC), and 481 small molecules in 1,001 cells lines from the Genomics of Therapeutics Response Portal (CTRP). Pearson correlation outputs adjusted to the false discovery rate are reported.

### Statistical methods

Comparison tests, overall survival analysis, univariate Cox regression, and the Kaplan–Meier method were performed using STATA/IC (version 16.1; STATA, College Station, TX; Computing Resource Center, Santa Monica, CA).

## Results

**FIGURE 1:**
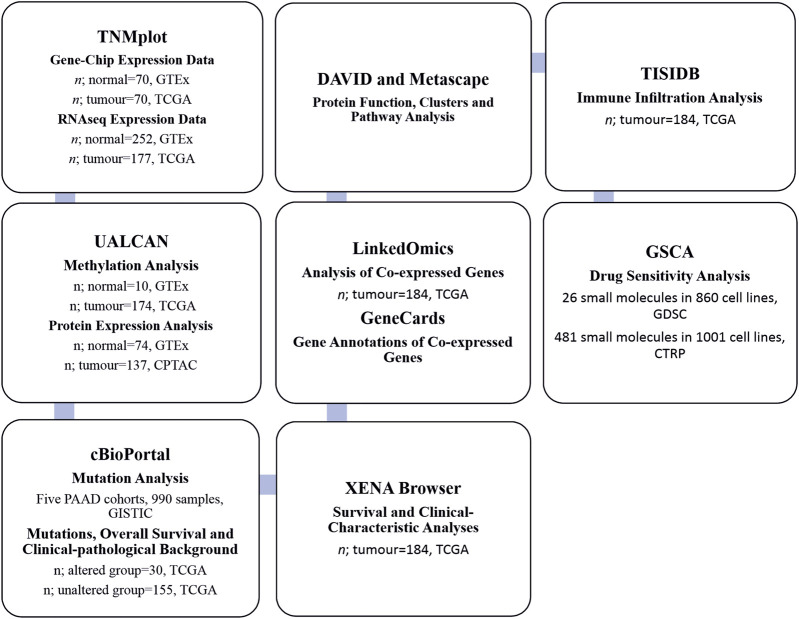
Flowchart of the search strategy and sample numbers of the datasets.

Gene, mRNA, and protein expression analyses, and the methods with sample numbers are shown in the workflow diagram (Figure 1).

### PAAD is associated with LAMA3, LAMB3, and LAMC2 upregulation at mRNA and protein levels

**FIGURE 2:**
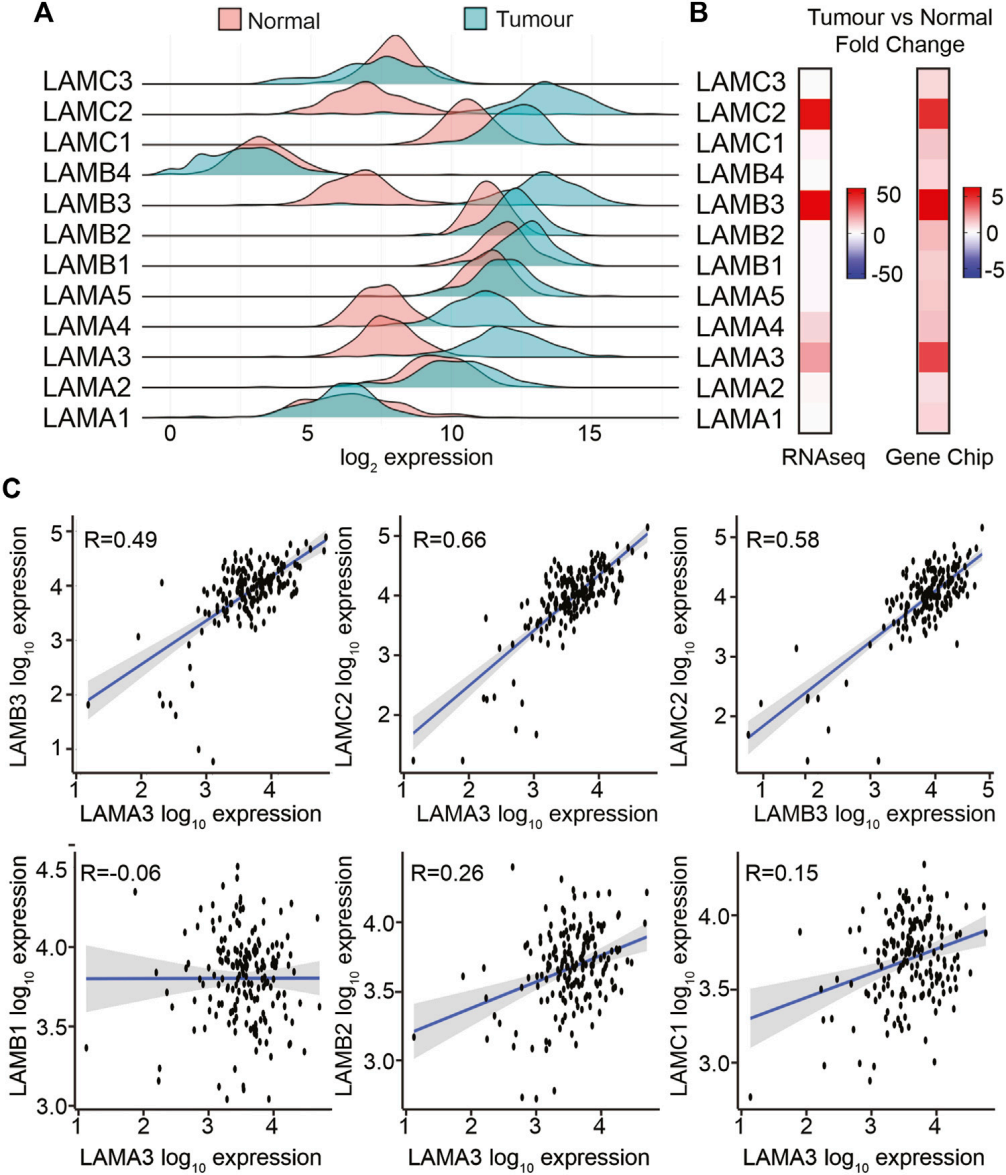
LAMA3, LAMB3, and LAMC2 mRNA expression levels are increased in PAAD. **(A)** Comparison of transcript levels of LM family members in normal (*n* = 252) and tumour tissues (*n* = 177) from RNA-seq TCGA data. Expression was normalised, and counted mean expression is shown as log_2_ fold changes. **(B)** Heatmaps comparing the mean fold difference between normal and PAAD tumour samples in RNA-seq (left) or gene chip (right) datasets. **(C)** Expression correlation between LAMA3 with LAMB3, LAMC2, LAMB1, LAMB2, and LAMC1, and LAMB3 with LAMC2.

The TNMPlot (https://tnmplot.com/) online tool was used to compare laminin transcript abundance in RNAseq data between normal pancreas from the GTEx dataset and PAAD tumour tissues from TCGA dataset (n; normal = 252, tumour = 177, Figures 2A, B; Supplemental Material S1A, S1B). Most laminin genes showed increased expression in the tumour tissue. However, LAMA1 was not statistically significantly different between groups, while LAMC3 and LAMB4 showed slight decreases in expression in tumour samples. It should be noted that LAMB4 is considered a pseudogene, and its mRNA abundance was much lower than all the other LM genes both in normal and in PAAD tissues (Figure 2A). The largest expression differences between tumour and normal samples were observed for LAMA3 (mean fold change; mfc = 21.7, *p* = 2.1 × 10^−61^), LAMB3 (mfc = 55.3, *p* = 7.9 × 10^−60^), and LAMC2 (mfc = 51.0, *p* = 3.8 × 10^−61^) transcripts.

To triangulate the RNAseq data, gene chip data were also analysed in the same way, comparing paired pancreatic tumours and adjacent normal tissues using the same online tool (*n* normal = 70, tumour = 70) (Figure 2B; Supplemental Material S1B). In the gene chip data, LAMA3, LAMB3, and LAMC2 again showed the greatest fold increase in the tumour tissue, at least 0.4-fold increased at *p* < 0.001, whereas changes in LAMA1, LAMA5, LAMB4, and LAMC3 did not reach statistical significance.

The protein products from LAMA3, LAMB3, and LAMC2 (LMα3, LMβ3, and LMγ2, respectively) heterotrimerise to form LM332; therefore, we anticipated co-regulation of these transcripts. Analysis of a within-sample correlation between the transcript abundance in tumours identified a correlation between LAMA3 and LAMB3 in both the RNAseq and gene chip data (Figure 2C RNAseq, Supplemental Material S2A), Spearman Rho: r = 0.49 RNA-seq, r = 0.82 gene chip, *p* < 0.01, and a stronger correlation between LAMA3 and LAMC2 (r = 0.66 RNA-seq, r = 0.67 gene chip, *p* < 0.01), and between LAMB3 and LAMC2 (r = 0.58 RNA-seq, r = 0.66 gene chip, *p* < 0.001). LAMA3-derived proteins are also capable of heterotrimerising with the proteins expressed from LAMB1 or LAMB2 and LAMC1 to produce LM311 or LM321, respectively (Copp et al., 2011; Aumailley, 2021). However, there was almost no correlation between LAMA3 and these genes (LAMA3 with LAMB1 r = −0.006, RNAseq and r = 0.12 gene chip, LAMA3 with LAMB2 r = 0.26 RNAseq and r = 0.16 gene chip, and LAMA3 with LAMC1 r = 0.15 RNAseq and r = 0.34 gene chip). Comparing all other laminin–laminin gene co-regulation in the RNAseq data (Supplemental Material S2B), a similar strength positive association was revealed between LAMA4, LAMB1, and LAMC1 as for LM332 (LAMA4 with LAMB1 r = 0.63, LAMA4 with LAMC1 r = 0.53, and LAMB1 with LAMC1 r = 0.45). No other correlations were above r = 0.5 or below r = −0.5.

**FIGURE 3:**
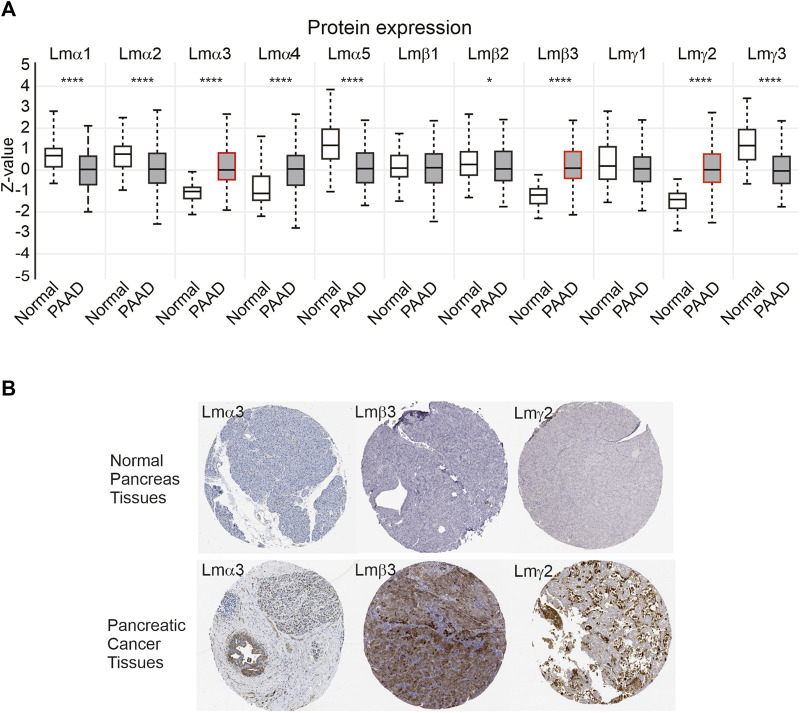
LM α3, β3, and γ2 protein levels are increased in PAAD samples. **(A)** Z-score comparison of normal (*n* = 74) and primary PAAD tumours (*n* = 137) from the CPTAC dataset, for each LM chain. Boxes represent 25th–75th percentile whisker to maximum and minimum and, line at median. Red outlines highlight LMα3, LMβ3, and LMγ2. Differences between normal and PAAD samples reach statistical significance at *p* < 0.001 for LMα1, LMα2, LMα3, LMα4, LMα5, LMβ3, LMγ2, and LMγ3. *p < 0.05, **p < 0.01, ***p < 0.001, and ****p < 0.0001. **(B)** Representative immunohistochemistry images of anti-LMα3, LMβ3, or LMγ2 in normal and pancreatic cancer specimens from the Human Protein Atlas (https://www.proteinatlas.org).

As a third mechanism to examine changes in the laminin expression, the protein data from CPTAC were analysed using the UALCAN platform (Figure 3A). Consistent with the transcript data, the greatest confidence in observed changes was with the LM332 proteins with higher expression in the primary tumour samples compared with control tissues for the LMα3 protein (median Z-score difference between tumour and control of 1.1, *p* = 2.9 × 10^−30^), LMβ3 (Z-score difference 1.3, *p* = 1.1 × 10^−33^), and LMγ2 (Z-score difference 1.41, *p* = 6.4 × 10^−36^). Other notable differences were a slight reduction in the LMα5 and LMγ3 protein expression in PAAD (Z-score differences of LMα5: 0.64, *p* = 4.6 × 10^−13^; LMγ3 1.22, *p* = 5 × 10^−13^) and the upregulation of LMα4 (Z difference 1.16, *p* = 1.8 × 10^−6^). The very small reductions in the expression of LMα1 and LMα2 in PAAD relative to normal samples also reached statistical significance at α of 0.01, whereas LMβ1, LMβ2, and LMγ1 differences did not reach this threshold (LMα1 *p* = 1.8 × 10^−8^, LMα2 *p* = 9 × 10^−8^, LMβ1 *p* = 0.46, LMβ2 *p* = 0.048, and LMγ1 *p* = 0.11). No products in this dataset mapped to LMβ4 were consistent with its pseudogene status and low transcript abundance. In immunohistochemistry, images deposited in the human tissue atlas (https://www.proteinatlas.org), LMα3, LMβ3, and LMγ2 were scored by the pathologists as “not detected” in the pancreatic endocrine tissue, normal tissue, or medium, or those not detected in the exocrine glandular normal tissue, however, were frequently detected with high expression in PAAD specimens (Figure 3B). For comparison, images from other laminin family entries in the human protein atlas are included in Supplemental Material S3.

### Clinical and pathological characteristics cause high expression of LAMA3, LAMB3, and LAMC2 in PAAD

**FIGURE 4:**
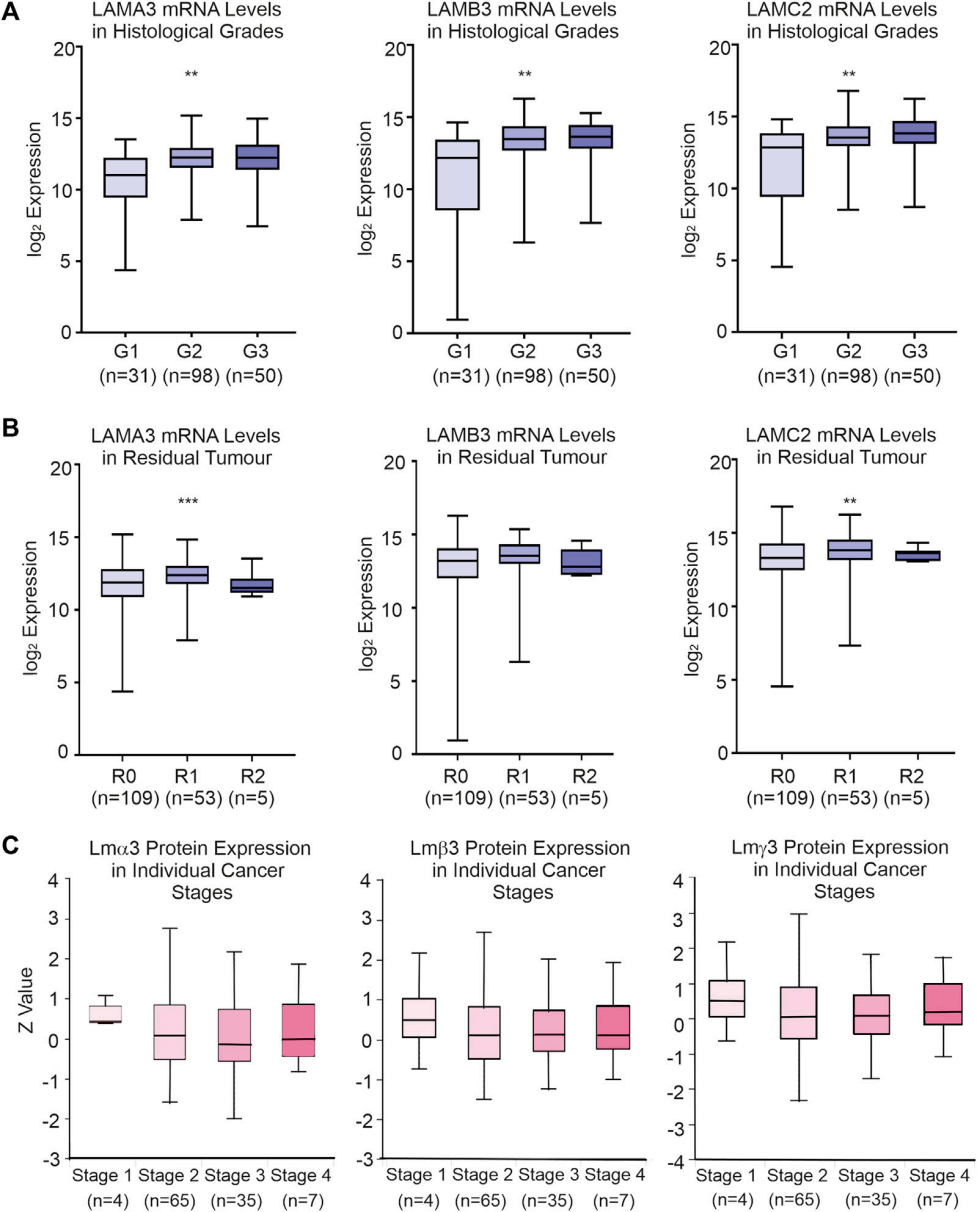
Association between clinical-pathological variables and LM332 mRNA and protein expression levels in patients with PAAD. **(A)** Log_2_ converted mRNA levels of LAMA3, LAMB3, and LAMC2 transcript abundance in histological grades (G1, grade 1; G2, grade 2; G3, grade 3). **(B)** Log_2_ converted mRNA levels of LAMA3, LAMB3, and LAMC2 transcript abundance in residual tumours (R0, residual 0; R1, residual 1; R2, residual 2). **(C)** Z-scores of protein levels of LMα3, LMβ3, and LMγ2 relative to the PAAD cancer stage. In each plot, boxes represent 25th–75th percentile whisker to maximum and minimum, and line at median; *p < 0.05, **p < 0.01, and ***p < 0.001.

Data from TCGA dataset [UCSC Xena (xenabrowser.net)] were used to identify associations between LM gene expression levels and clinical variables (Table 1; Supplemental Material S4). Patients with the upper third LAMA3 (*p* = 0.01), LAMB3 (*p* = 0.01) and LAMC2 (*p* = 0.01) transcript abundance were each associated with a higher histological grade, while high LAMA3 (*p* = 0.001) and LAMC2 (*p* = 0.01) were associated with the residual tumour stage (Figures 4A, B; Table 1). Other observations included a less robust correlation between LAMA3 with pathological stages (*p* = 0.04) and LAMB3 with male patients (*p* = 0.02) and with chronic pancreatitis history (*p* = 0.03). High mRNA expression of LAMB4 was only associated with the T-stage (Table 1). At the protein level, the biggest differences were between normal and tumour tissues, and no statistically significant differences were observed between any histological stages (Figure 4C).

**TABLE 1:** Association between high expression of LAMA3, LAMB3 and LAMC2 transcripts and clinicopathological features in the PAAD cohort. Chi-square and Fisher’s exact tests were used to compare the low expression (first tercile) and high expression (last tercile) groups for association with clinical features. Statistical significance was considered as *p* < 0.05. Fisher’s exact results shown with “*”.

		LAMA3			LAMB3			LAMC2		
	N/%	High N = 62	Low N = 61	*p*	High N = 62	Low N = 61	*p*	High N = 61	Low N = 61	*p*
Age				0.66			0.66			0.37
<65	59 (48)	34 (55)	31 (51)		34 (55)	31 (51)		33 (54)	28 (46)	
≥65	63 (52)	28 (45)	30 (49)		28 (45)	30 (49)		28 (46)	33 (54)	
Sex				0.92			0.02*			0.72
Female	56 (46)	27 (44)	26 (43)		22 (35)	34 (56)		27 (44)	29 (48)	
Male	66 (54)	35 (56)	35 (57)		40 (65)	27 (44)		34 (56)	32 (52)	
Smoker				0.49			0.97			0.92
Yes	49 (49)	27 (54)	24 (47)		27 (52)	23 (52)		25 (51)	24 (50)	
No	52 (51)	23 (46)	27 (53)		25 (48)	21 (48)		24 (49)	24 (50)	
Alcohol				0.88			0.65			1
Yes	75 (65)	36 (61)	34 (60)		36 (63)	33 (59)		35 (60)	35 (60)	
No	41 (35)	23 (39)	23 (40)		21 (37)	23 (41)		23 (40)	23 (40)	
Diabetes				0.23			0.41			0.31
Yes	31 (32)	10 (20)	15 (30)		12 (23)	14 (30)		9 (18)	13 (27)	
No	67 (68)	41 (80)	35 (70)		40 (77)	32 (70)		41 (82)	36 (73)	
Pancreatitis				1			0.03*			0.50
Yes	10 (10)	4 (8)	3 (6)		8 (16)	1 (2)		6 (13)	4 (8)	
No	86 (90)	45 (92)	45 (94)		42 (84)	45 (98)		42 (87)	44 (92)	
Family history of cancer				0.65			0.10			0.91
Yes	47 (59)	21 (54)	23 (59)		14 (39)	21 (58)		21 (53)	21 (54)	
No	32 (41)	18 (46)	16 (41)		22 (61)	15 (42)		19 (47)	18 (46)	
Anatomic subdivision				0.64			0.71			0.54
Head of the pancreas	92 (75)	47 (76)	44 (72)		46 (74)	47 (77)		46 (75)	43 (70)	
Other	30 (25)	15 (24)	17 (28)		16 (26)	14 (23)		15 (25)	18 (30)	
Radiation therapy				0.62			0.79			0.49
Yes	28 (25)	15 (26)	18 (31)		16 (28)	17 (30)		14 (25)	17 (30)	
No	84 (75)	42 (74)	41 (69)		42 (72)	40 (70)		43 (75)	39 (70)	
Residual tumour				0.001*			0.18			0.01*
R0	76 (66)	31 (53)	41 (77)		35 (64)	43 (76)		31 (56)	43 (80)	
R1	35 (30)	27 (47)	9 (17)		19 (34)	11 (19)		24 (44)	11 (20)	
R2	4 (4)	0 (0)	3 (6)		1 (2)	3 (5)		-	-	
Histologic grade				0.01*			0.01*			0.01*
G1	25 (21)	5 (8)	18 (30)		4 (6)	18 (30)		6 (10)	18 (31)	
G2	64 (53)	35 (56)	25 (42)		37 (60)	28 (47)		33 (54)	28 (47)	
G3	31 (25)	22 (36)	15 (25)		21 (34)	13 (21)		22 (36)	11 (19)	
G4	1 (1)	0 (0)	2 (3)		0 (0)	1 (2)		0 (0)	2 (3)	
Pathologic stage				0.04*			0.10			0.07
Stage I	16 (13)	2 (3)	10 (17)		4 (7)	12 (20)		4 (7)	13 (22)	
Stage II	96 (80)	55 (89)	47 (79)		54 (87)	45 (76)		52 (85)	43 (73)	
Stage III	4 (3)	2 (3)	1 (2)		2 (3)	1 (2)		2 (3)	1 (2)	
Stage IV	5 (4)	3 (5)	1 (2)		2 (3)	1 (2)		3 (5)	2 (3)	
T-stage				0.15			0.32			0.14
T1	5 (4)	0 (0)	3 (5)		2 (3)	4 (7)		1 (2)	3 (5)	
T2	17 (14)	6 (9)	10 (17)		6 (10)	11 (18)		5 (8)	12 (20)	
T3	96 (80)	55 (89)	45 (76)		53 (85)	43 (73)		54 (88)	43 (73)	
T4	3 (2)	1 (2)	1 (2)		1 (2)	1 (2)		1 (2)	1 (2)	
N-stage				0.18			0.91			0.29
N0	33 (28)	12 (20)	17 (30)		19 (31)	18 (32)		16 (27)	20 (36)	
N1	84 (72)	49 (80)	39 (70)		42 (69)	38 (68)		44 (73)	36 (64)	

As changes in the laminin gene expression have been associated with changes to the immunophenotype of a tumour in different contexts (Diao and Yang, 2021), we also queried the LAMA3, LAMB3, and LAMC2 genes in the TIMER2.0 database to investigate their relationship with tumour-infiltrating lymphocytes (Supplemental Material S5). There was no strong correlation with any immunomodulatory cell types; however, some very weak associations were observed between LAMA3 and infiltration of B cells (r = 0.24. *p* = 1.9 × 10^−3^) and dendritic cells (r = 0.18, *p* = 1.6 × 10^−2^), LAMB3 with B cells (r = 0.20, *p* = 1.1 × 10^−2^), and LAMC2 with dendritic cells (r = 0.19, *p* = 1.1 × 10^−2^). TISIDB (hku.hk) was also used to examine the co-regulation of immune-regulating genes (Supplemental Material S6). LAMA3, LAMB3, and LAMC2 mRNA levels had a weak negative correlation with immunoinhibitory genes including *ADORA2* and *CD160*, immunostimulatory genes including *CXCL12*, *KLRK1*, and *TNFRSF14*, and MHC molecules such as HLA-DPB1 in PAAD.

### High LM332 mRNA expression predicts worse patient survival outcomes in PAAD

**FIGURE 5:**
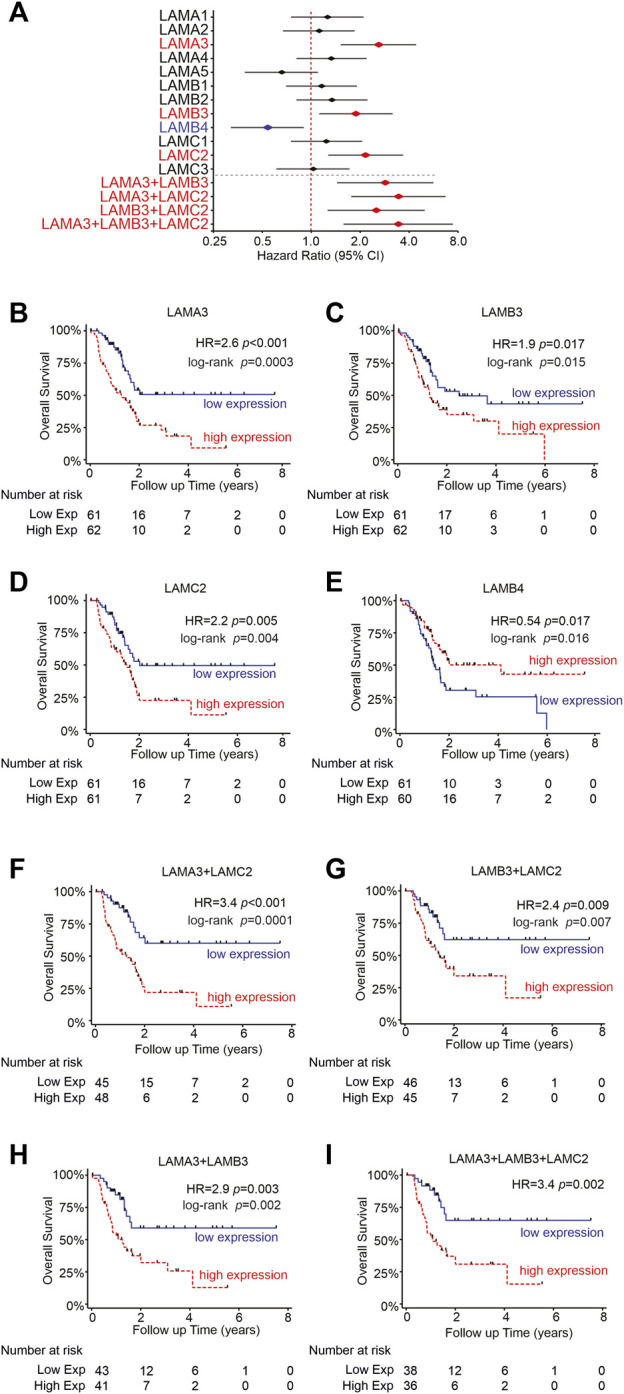
High LM332 gene expression predicts poor patient survival rates in PAAD. **(A)** Forest plot of hazard ratios (diamond) with 95% confidence intervals for overall survival, comparing the upper third RNA expression level of each gene or gene combinations in PAAD patients versus the lower third expression levels. Positive numbers indicate worse survival rates in the high-expression group. Red text and symbols or blue text and symbols indicate statistically significant (*p* < 0.05) worse outcomes for high- or low-expression groups, respectively. **(B–E)** Kaplan–Meier plots for individual genes, comparing upper and lower terciles and **(F–I)** Kaplan–Meier plots for indicated pairs/triplet of genes. HR = hazard ratio with *p*-values calculated using Cox regression.

Next, we asked if the laminin expression predicts the survival outcome of PAAD patients. Expression data for each laminin gene were trichotomized, and overall survival (OS) times between the upper and lower thirds were compared (Figures 5A–D). Patients with high expression of LAMA3, LAMB3, or LAMC2 were each associated with worse OS, with hazard ratios (HR) of 1.9–2.6 (Figures 5A–D, LAMA3 HR = 2.6, 95% CI 1.5-4.4, *p* < 0.0001; LAMB3 HR = 1.9, 95% CI 1.1-3.2, *p* = 0.017; LAMC2 HR = 2.15, 95% CI 1.8-6.7, *p* = 0.005). In contrast, the high-LAMB4 expression group was associated with better OS (Figures 5A, E, HR = 0.5, 0.3−0.9, *p* = 0.017). Other laminin genes did not show any statistically significant association with survival outcomes (Figure 5A).

Although LAMA3, LAMB3, and LAMC2 expression levels frequently correlated with one another, not every sample shows upregulation of all three genes, as demonstrated in Figure 2. Anticipating that functional differences may require all three genes to be concomitantly upregulated to increase LM332 heterotrimer production, we assessed the survival data further, comparing samples where pairs or triplets of the genes were both/all upregulated (Figures 5A, F–I). In support of our hypothesis, high expression of at least two members of each pair of genes was associated with higher HRs than using individual genes alone. The strongest predictive value coming from LAMA3 with LAMC2 (Figures 5A, F–H), LAMA3+LAMB3 HR = 2.9, 95% CI 1.4-5.6, *p* = 0.003; LAMA3+LAMC2 HR = 3.4, 95% CI 1.8-6.7, *p* = 0.0001; LAMB3+LAMC2 HR = 2.5, 95% CI 1.3-5.0; *p* = 0.009. Combining all three genes LAMA3+LAMB3+LAMC2 also yielded higher HR than any individual gene (Figures 5A, I, HR = 3.43, 95% CI 1.6-7.4, *p* = 0.002) but did not add any additional value beyond the pairing of LAMA3+LAMC2. These findings suggest using LAMA3 and LAMC2 together as prognostic biomarkers for PAAD patients.

### LAMA3, LAMB3, and LAMC2 gene mutations are associated with the clinical stage but not with patient survival

**FIGURE 6:**
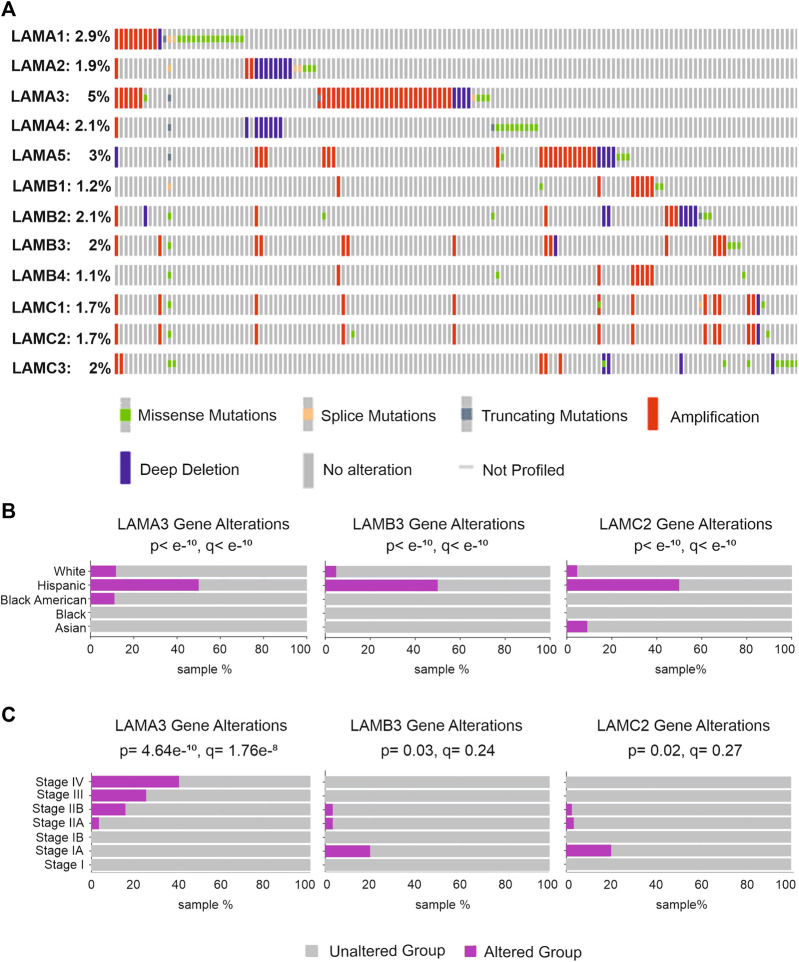
Genetic alteration rates in laminin genes and relationship with clinicopathological background in patients with pancreatic tumours. **(A)** LM family gene alterations in pancreatic cancer; **(B,C)** pancreatic cancer patients with LAMA3, LAMB3, and LAMC2 alterations and the relationship between alterations and patient ethnicity **(B)** or detailed disease stages **(C)**.

Gene mutation analysis of five different pancreatic cancer studies, including PAAD and PDAC data (990 samples), was analysed using the cBioPortal online tool (www.cbioportal.org) (Figure 6A). LAMA3 had the highest mutation rate of all laminin genes (5%), with alterations including amplifications, missense mutations, splice mutations, deep deletions, and truncating mutations. LAMA5 (3%) and LAMA1 (2.9%) showed the next highest mutation frequency. LAMB3, LAMC2, and LAMB4 had alteration rates of 2%, 1.7%, and 1.1%, respectively. Gene alterations of LAMA3, LAMB3, and LAMC2 were each disproportionately associated with Hispanic patients (Figure 6B). Histopathologically, LAMA3 alterations were associated with patients with later stages of PAAD (*p* = 4.6 × 10^−10^, q = 1.7 × 10^−8^), whereas associations between LAMB3 and LAMC2 and the disease stage were less robust but suggest a slight association with early disease (*p* = 2.8 × 10^−4^, q = 4.4 × 10^−3^; *p* = 0.02, q = 0.27) (Figure 6C). There were no statistically significant effects of laminin gene-level changes on the overall survival of PAAD patients (Supplemental Material S7).

### Altered regulatory mechanisms are associated with LM332 dysregulation

**FIGURE 7:**
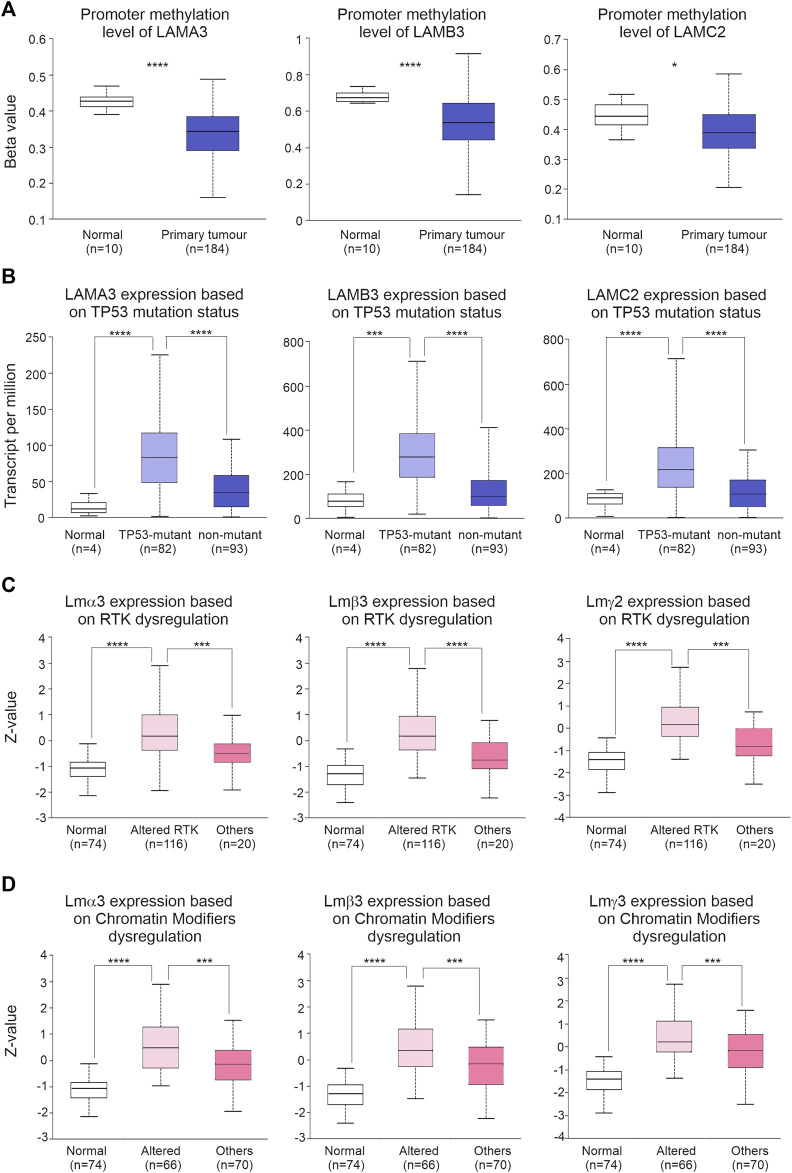
Dysregulated regulatory mechanisms are associated with LM332 alterations in PAAD. **(A)** Promoter methylation levels of LAMA3, LAMB3, and LAMC2 in normal and PAAD tumour tissues; data shown as β-values. **(B)** Transcript abundance of LAMA3, LAMB3, and LAMC2 in normal, TP-53-mutant PAAD, and non-TP53-mutant PAAD patients; data shown as transcript per million. **(C)** Protein abundance of LMα3, LMβ3, and LMγ2 in normal, PAAD with altered receptor tyrosine kinase (RTK), and PAAD with other alterations; data shown as the Z-value. **(D)** Protein abundance of LMα3, LMβ3, and LMγ2 in normal pancreatic samples, in PAAD with altered chromatin modifiers dysregulation, and in PAAD with other alterations; data shown as the Z-value. In each plot, boxes represent 25th–75th percentile whisker to maximum and minimum, and line at median; *p < 0.05, **p < 0.01, ***p < 0.001, and ****p < 0.0001.

We next explored potential drivers for the changes in LM332 transcript abundance. Promoter methylation was lower in PAAD than normal pancreatic tissues for all three genes (LAMA3 normal β:0.43, tumour β:0.34, *p* = 2.8 × 10^−9^; LAMB3 normal β:0.65, tumour β:0.53, *p* = 8.6 × 10^−7^; and LAMC2 normal β:0.44, tumour β:0.38, *p* = 0.04) (Figure 7A). All three transcript levels of LM332 were higher in TP53-mutant PAAD tissues than those in normal pancreatic and non-TP53 mutant PAAD tissues (transcript per million, normal: 11.6, TP53-mutant: 83.2, TP53-nonmutant: 34.5, normal-vs-TP53-mutant *p* = 2 × 10^−8^, normal-vs-TP53-nonmutant *p* = 0.014, TP53-mutant-vs-TP53-nonmutant *p* = 2.1 × 10^−7^, LAMB3 normal: 77.6, TP53-mutant: 278.6, and TP53-nonmutant: 98.6; normal-vs-TP53-mutant *p* = 0.0017, normal-vs-TP53-nonmutant *p* = 0.29, and TP53-mutant-vs-TP53-nonmutant *p* = 2.8 × 10^−10^; LAMC2 normal: 89.7, TP53-mutant: 216.7, TP53-nonmutant: 103.8; and normal-vs-TP53-mutant *p* = 7.5 × 10^−5^, normal-vs-TP53-nonmutant *p* = 0.37, and TP53-mutant-vs-TP53-nonmutant *p* = 3.6 × 10^−8^) (Figure 7B). At the protein level, all three proteins were higher in RTK-dysregulated PAAD tumours than those in normal pancreatic and other PAAD tumours (LMα3 protein normal Z:-1.06, altered RTK Z: 0.16, others Z:-0.5, normal-vs-altered RTK *p* = 7.8 × 10^−30^, normal-vs-others *p* = 6.8 × 10^−4^, altered RTK-vs-others *p* = 9.4 × 10^−4^, LMβ3 protein normal Z:-1.3, altered RTK Z: 0.16, and others Z:-0.77; normal-vs-altered RTK *p* = 2.4 × 10^−34^, normal-vs-others *p* = 1 × 10^−3^, altered RTK-vs-others *p* = 1 × 10^−4^, and LMγ2 protein normal Z:-1.41, altered RTK Z:0.14, and others Z:-0.82; and normal-vs-altered RTK *p* = 1.3 × 10^−37^, normal-vs-others *p* = 6.4 × 10^−4^, and altered RTK-vs-others *p* = 1.4 × 10^−4^) (Figure 7C). Similarly, LM332 proteins were highly expressed in PAAD tumours with dysregulated chromatin modifiers LMα3 protein normal Z:-1.06, altered chromatin modifiers Z: 0.48, and others Z:-0.13; normal-vs-altered chromatin modifiers *p* = 5.7 × 10^−22^, normal-vs-others *p* = 1.1 × 10^−13^, altered chromatin modifiers-vs-others *p* = 5.3 × 10^−4^, LMβ3 protein normal Z:-1.3, altered chromatin modifiers Z: 0.34, and others Z:-0.17; normal-vs-altered chromatin modifiers *p* = 5.4 × 10^−24^, normal-vs-others *p* = 1.2 × 10^−15^, altered chromatin modifiers-vs-others *p* = 6.4 × 10^−4^, and LMγ2 protein Z:-1.41, altered chromatin modifiers Z: 0.2, and others Z:-0.16; and normal-vs-altered chromatin modifiers *p* = 1.8 × 10^−26^, normal-vs-others *p* = 4.7 × 10^−18^, and altered chromatin modifiers-vs-others *p* = 5 × 10^−4^ (Figure 7D).

### LAMA3, LAMB3, and LAMC2 co-regulated genes that of high expression predicts worse survival

**FIGURE 8:**
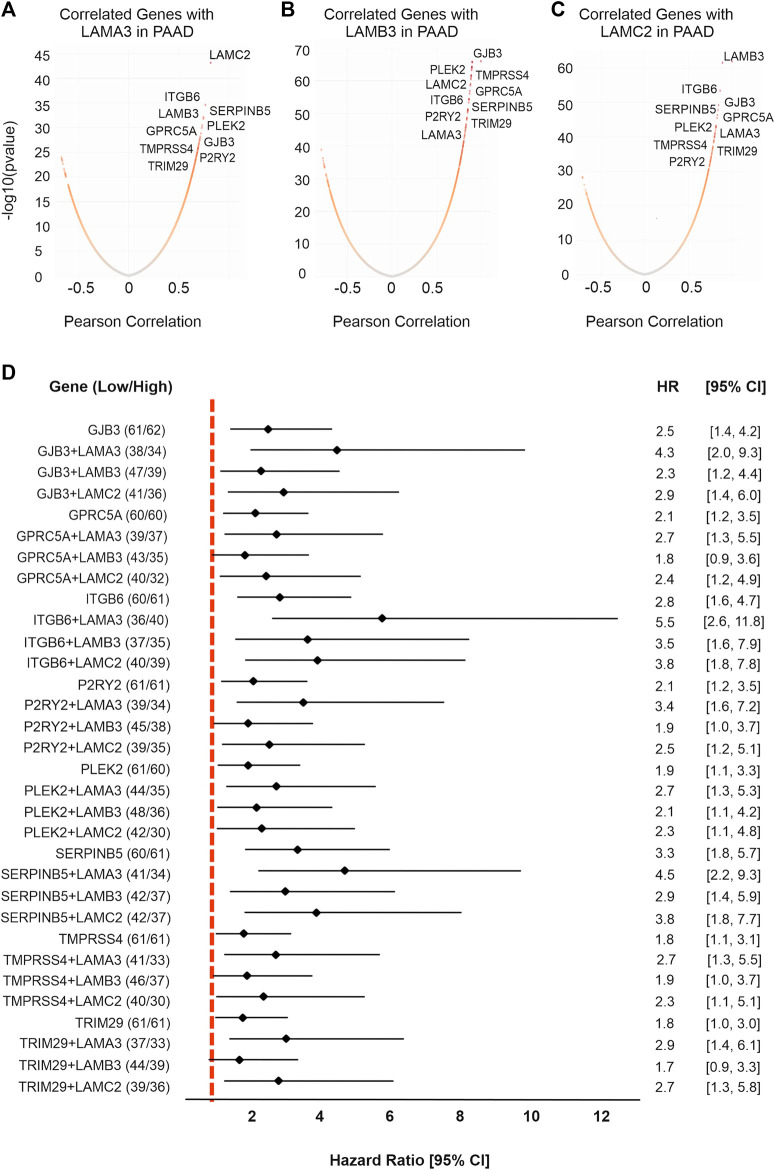
LM332 is co-expressed with eight hub genes which are associated with poor survival in PAAD patients. Co-expressed genes with **(A)** LAMA3, **(B)** LAMB3, and **(C)** LAMC2 were presented in volcano plots, expression levels converted to log_10_, and correlation shown as the Pearson correlation. **(D)** Forest plot of hazard ratios with 95% confidence intervals for overall survival comparing the upper third expression level of each gene or gene combinations in PAAD patients versus the lower third expression levels. Positive numbers indicate worse survival rates in the high-expression group.

We next analysed co-expressed genes using the LinkedOmics database (http://www.linkedomics.org/) and samples from patients with PAAD (Figures 8A–C; Supplemental Material S8). These analyses identified common genes that were co-expressed with LAMA3, LAMB3, and LAMC2 genes. Within this dataset were eight hub genes related to all three genes, namely, the integrin ITGB6, the serpin peptidase inhibitor SERPINB5, the actin and phosphatyl inositol phosphate-binding protein pleckstrin 2 PLEK2, G-protein receptor GPRC5A, gap junction channel protein GJB3, serine-type endopeptidase TMPRSS4, purinergic receptor P2RY2, and tripartite motif-containing protein 29 TRIM29. Other notable correlations were LAMA3 with ITGA2, protein tyrosine kinase receptor MET, and actin-binding TMOD3; LAMB3 and LAMC2 with LM332-binding integrin ITGB4 (Hamill et al., 2010), and with transmembrane serine protease 4 TMPRSS4. LAMA3, LAMB3, and LAMC2 all strongly negatively correlated (Pearson correlation < −0.6) with the sulphate transmembrane transporter SLC26A11, galactosyltransferase activity protein B3GNT1, GTP-binding protein RND2, nucleic acid-binding protein FXR2, coenzyme COQ10A, heme-binding protein CYB5D2, calmodulin and syntaxin-binding protein VAMP2, phosphoprotein phosphatase activity protein PDXP, C20orf132 and C12orf34, and electron transfer protein QDPR.

High expression of all eight co-expressed upregulated hub genes predicted poor OS in patients with PAAD (Figure 8D). Of these, SERPINB5 was the strongest predictor of outcomes, with a higher predictive value than the individual laminin-encoding genes, and a similar value to the combined LM332 signature (Figure 8D) (SERPINB5 HR = 3.3, *p* < 0.0001; ITGB6 HR = 2.81, *p* < 0.0001; PLEK2 HR = 1.97, *p* < 0.0001; GPRC5A HR = 2.15, *p* = 0.003; GJB3 HR = 2.50, *p* = 0.0004; TMPRSS4 HR = 1.85, *p* = 0.02; P2RY2 HR = 2.10, *p* = 0.005; and TRIM29 HR = 1.82, *p* = 0.021). Interestingly, the patients who had high LAMA3 and high expression of any one of the co-expressed genes had worse OS than those with low expression (Figure 8D). Of particular note, combining LAMA3 with ITGB6 yielded an HR of 5.6 (95% CI 2.6-11.9) and SERPINB5 increased to an HR of 4.6 (95% CI 2.2-9.3).

The top 50 co-expressed genes with LAMA3, LAMB3, and LAMC2, were entered in the DAVID online tool DAVID Functional Annotation Bioinformatics Microarray Analysis (ncifcrf.gov) for functional annotations including BP, MF, and CC (Supplemental Material S9A, S9B). As expected for basement membrane proteins, major enriched process terms included “cell adhesion” and “host-virus interaction;” component terms of “cell junction,” “BM,” and “ECM;” and functional terms of “receptor” and “integrin.” KEGG pathway analysis identified that 11 genes were associated with the KEGG term “PI3K-Akt signalling pathway,” which has been associated with LM332-mediated signalling (Zhang et al., 2019; Jung et al., 2018) (Supplemental Material S9A). The Metascape database (https://metascape.org/) was used to explore protein–protein interactions of the genes co-expressed with LM332 in PAAD, the interaction network and Minimal Common Oncology Data Elements (MCODE) components enrichment of the “ECM-receptor interaction,” “VEGFA-VEGFR2 signalling pathway,” and “gap junction assembly” (- log10(*p*) = 8.8″) (Supplemental Material S9B.

### High LAMA3, LAMB3, and LAMC2 expression levels are associated with sensitivity to EGFR inhibitors

**FIGURE 9:**
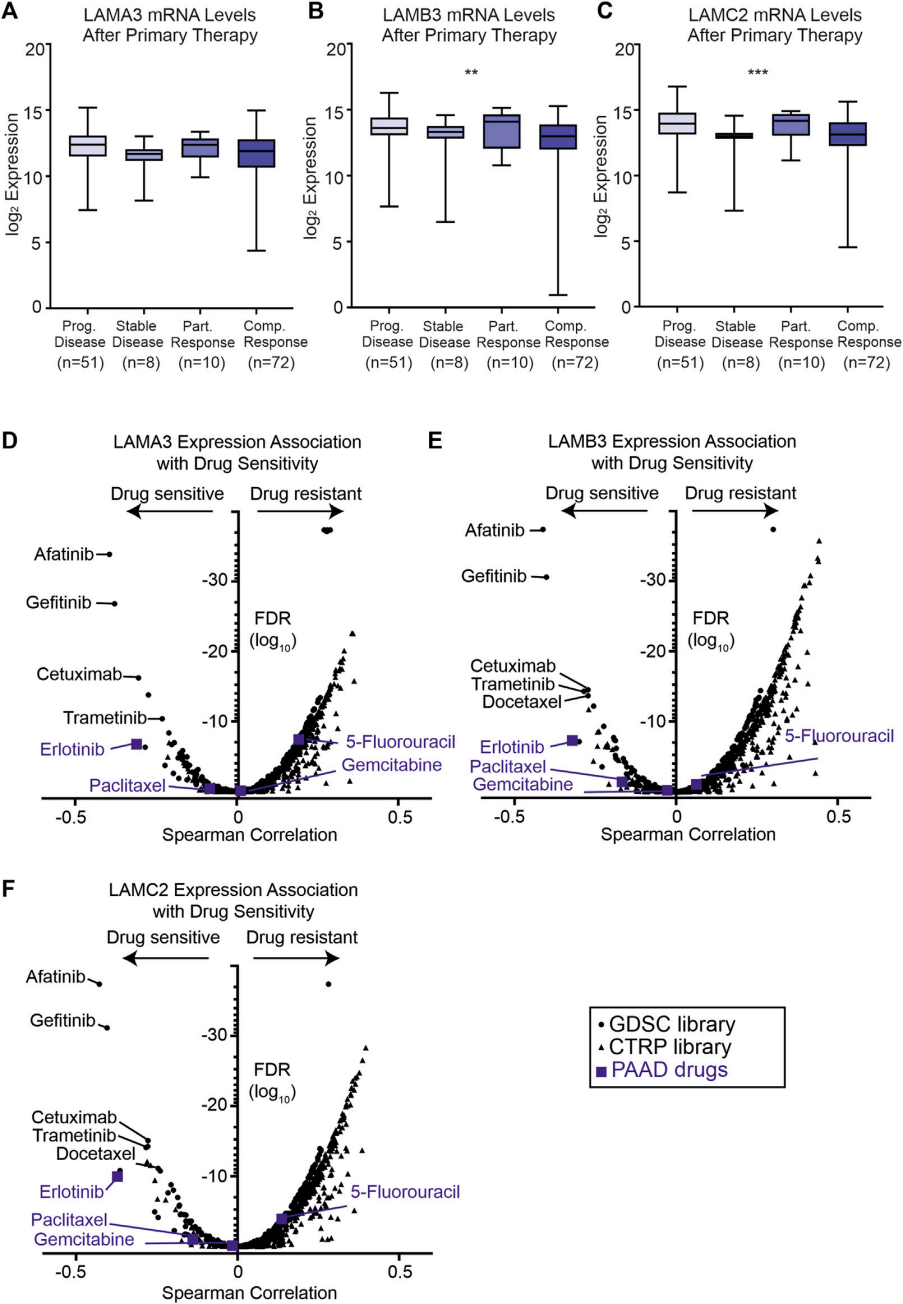
High LAMA3, LAMB3, and LAMC2 expression are related to primary therapy and sensitivity to EGFR inhibitors. **(A–C)** Z scores of protein levels of LMα3 **(A)**, LMβ3 **(B)**, and LMγ2 **(C)** in samples after primary therapy then segregated based on clinical outcome into either progressive disease, stable disease, partially response or complete response, *p < 0.05, **p < 0.01, ***p < 0.001, and ****p< 0.0001. **(D–F)** Volcano plots of Spearman correlation coefficients between LAMA3 **(D)**, LAMB3 **(E)**, LAMC2 **(F)** and sensitivity to the tested compound plotted against the false discovery rates (FDR). Data were generated from drug sensitivity screens in cancer lines as a combination of Genomics of Drug Sensitivity in Cancer (GDSC, squares) and the Therapeutics Response Portal (CTRP, triangles) screens, accessed using the GSCA platform. Commonly used pancreatic adenocarcinoma therapeutics are coloured blue.

As high expression of LM332 was associated with poor survival rates, we next asked if the expression of these genes correlated with response to primary therapy (Figures 9A–C). Patients with the progressive disease had higher expression of LAMB3 (*p* = 0.01) and LAMC2 (*p* = 0.001) than those with the stable disease with the trend for LAMA3 being the same but not statistically significant (*p* = 0.13). Additionally, partial responders to therapy had higher expression of these genes than those with complete responses (Figures 9A–C).

To investigate whether patient stratification by their laminin gene expression profiles could aid therapeutic decisions, two drug sensitivity screens using cancer lines were interrogated using the GSCA platform (GSCA: Gene Set Cancer Analysis (hust.edu.cn) (Figures 9D–F). As expected from their correlated expression profiles, similar compounds were found to be highly effective against cell lines with high LAMA3, LAMB3, or LAMC2 expression (Figures 9D–F).

The frontline PAAD drugs paclitaxel and gemcitabine were not associated with strong sensitivity or resistance relative to these laminin expression levels, whereas high LAMA3 or LAMC2 exhibited a low level of resistance to 5-fluorouracil (LAMA3 Spearman rho 0.19, FDR 3 × 10^−8^; LAMC2 0.14, FDR 0.0001). In contrast, high expression of all three genes was associated with increased sensitivity to the epidermal growth factor (EGFR) tyrosine kinase inhibitor erlotinib (LAMA3 -0.31 FDR 1.7 × 10^−7^, LAMB3 -0.32 FDR 5.1 × 10^−8^, and LAMC2 -0.37 1.0 × 10^−10^). For other compounds in clinical use, the greatest sensitivity was observed for three other EGFR inhibitors; afatinib, gefitinib, and cetuximab, for the mitogen-activated kinase inhibitor trametinib, and, for LAMB3 and LAMC2, the tubulin-targeting taxol derivative docetaxel. High expression of all eight of the co-expressed genes was also associated with sensitivity to the same panel of compounds (Supplemental Material S10).

## Discussion

The findings presented here show that LAMA3, LAMB3, and LAMC2 are frequently upregulated at the gene and protein levels in PAAD tumours compared with controls and that these increases are related to worse survival outcomes. Moreover, these expression changes likely reflect a combination of disrupted RTK signalling, TP53 functionality, and chromatin remodelling, leading to transcriptional changes. Importantly, combining these data with drug sensitivity findings suggests that a subset of mainline PAAD therapeutics may not be the optimal choice for patients with elevated LM332. Therefore, screening and stratification based on the LM332 expression may be valuable for the development of personalised therapeutic intervention strategies.

We entered this project to examine the entire laminin family and their interplay. We anticipated that opposing changes or ratio-metric differences between the chains would provide deeper insights into how tumours progress and change. It was, therefore, quite surprising to find that it was only the LM332 genes/protein that was consistently and robustly associated with PAAD, which were associated with clinical features, and not the other major laminin chains. The finding that LM332 predicts outcomes is itself not surprising; the LM332 expression is related to poor 5- and 10-year OS in breast tumours, and poor 10-year OS in triple-negative breast cancer (Carpenter et al., 2018), and small cell lung cancer shows the association with shorter OS (Niki et al., 2001). Expression of the LAMC2 chain is associated with worse survival in oesophageal squamous cell carcinomas, oral squamous cell carcinomas, and colorectal carcinoma (Yamamoto et al., 2001; Fukai et al., 2005; Gasparoni et al., 2007; Aoki et al., 2023; Katoh et al., 2002). However, it should be noted that there is context specificity as high expression of LAMA3 in ovarian cancer was associated with better OS, recurrence-free survival, and 5-year survival rates (Tang et al., 2019). Prior studies have also demonstrated a relationship between survival and the sub-chains of LM332 in PAAD/PDAC. However, one of the key findings described here is that the HRs become larger, i.e., effect sizes are greater, when two or three of the LM332 genes are combined. Moreover, combining LAMA3 with any of their co-expressed genes further improved their predictive power. We cannot infer from these data alone whether the combinatorial effect reflects the expression of the LM332 protein driving tumour progression or are a consequence of shared dysregulation, but these findings suggest that adding LAMA3 to existing biomarkers may improve their functionality.

Among the genes positively co-expressed with LAMA3, LAMB3, and LAMC2, there are important proteins which may change cancer cell behaviour and cancer progression in different tumours. Integrins are molecules which bind ECM and regulate cell proliferation, migration, invasion, and survival. They are involved in many aspects of cancer progression (Desgrosellier and Cheresh, 2010). Higher mRNA levels of ITGB6 and ITGB4 were detected in pancreatic cancer patients with higher histologic grades. Moreover, the overexpression of these genes is associated with the upregulation of the notch signalling pathway (Zhuang et al., 2020). GJB3, TMPRRS4, GPRC5A, and TRIM29 were all identified previously, as upregulated in pancreatic cancer tissues (Kong et al., 2020). Of these, GPRC5A has been indicated as being capable of promoting proliferation and metastasis via regulating EMT (Qian et al., 2021), TMPRRS4 is considered an inhibitor of apoptosis and able to increase cell proliferation by inducing the ERK1/2 signalling pathway (Gu et al., 2021). TRIM29 has been shown to regulate cancer-stem cell-like profiles in pancreatic carcinomas (Sun et al., 2020). An increased SERPINB5 level has been associated with metastasis in PDAC (Mardin et al., 2010). Downregulation of PLEK2 by miRNA displayed inhibited the PI3K/AKT pathway and self-renewal in pancreatic cancer stem cells and promoted apoptosis (Yang et al., 2021). We are not aware of studies at this time analysing the direct association between the purinergic receptor P2RY2 and PAAD; this receptor is known to upregulate selinexor, which, in turn, activates PI3K/AKT signalling in acute myeloid leukaemia (Lin et al., 2022). It is well-known that abnormal activation of the PI3K-Akt signalling pathway is related to malignancy, there is also a relationship between PI3K and receptor tyrosine kinases upstream of PI3K (Noorolyai et al., 2019), and furthermore, the KEGG pathway analysis of LM332-co-expressed gene revealed that a correlation with PI3K-Akt signalling pathway genes and numerous *in vitro* studies have linked LM332 with PI3K activation (Yazlovitskaya et al., 2015; Zhang et al., 2019; Sui et al., 2022). Therefore, we suggest that the overexpression of LM332 in PAAD tumours represents a more widespread upregulation of genes promoting tumorigenesis poor outcomes.

One of the potential reasons for worse outcomes for PAAD patients with elevated LM332 expression could have been dysregulation of immune regulation associated with changes to the BM in the tumour environment. Laminin expression changes have been associated with this type of dysregulation previously in different cancers. High levels of LMγ2 were associated with fewer T cells and lymphocyte infiltration in oesophageal cancer or non-small cell lung cancer (Li et al., 2021). The LAMA5 expression modulated immune regulation by altering T-cell behaviours in lymph nodes (Li et al., 2020). LAMC2 was associated with CD8^+^ T-cell and B-cell infiltration in head and neck cancer (Jiang et al., 2020). However, here, all detected associations were lower than 0.4. There were some specific immune-related genes where high expression of LAMA3, LAMB3, and LAMC2 genes correlated with increased expression, and of these, the immuno-inhibitor genes PVRL2 and NT5E, and chemokines CCL20 and CXCL5. PVRL2 is overexpressed in multiple types of cancers, and the blockage of PVRL2 and PVRIG genes increases T-cell function (Whe et al., 2020). The immune-stimulator gene *NT5E* is related to poor survival in gastric cancer, and silencing of NT5E suppresses proliferation, invasion, and migration (Hu et al., 2019). However, chemokine CCL20 is upregulated in patients with pancreatic cancer and related to advanced T categories (Rubie et al., 2010), and high expression of CXCL5 is associated with shorter OS in hepatocellular and cholangiocarcinoma (Hu et al., 2018). Therefore, although findings overall suggest that LM332 effects are not related to immune dysfunction, we cannot rule out entirely that some of the co-regulated genes influence tumour progression. Additionally, increased immuno-inhibitor genes *PVRL2* and *NT5E* would be the key genes inhibiting immune response in patients with PAAD.

Importantly, these analyses identified that the high expression of LM332 genes was not associated with any strong sensitivity to some of the most commonly used pancreatic cancer-targeting drugs gemcitabine, paclitaxel, and docetaxel but increased sensitivity to other well-characterised chemotherapeutic drugs, gefitinib, erlotinib, afatinib, and cetuximab. Gefitinib and erlotinib are cancer drugs which have a common chemical backbone structure, and they are commonly used as EGFR-tyrosine kinase inhibitors in lung carcinoma (Tarceva et al., 2005; Xu et al., 2010; Chen et al., 2013). There were also three more tyrosine kinase drugs negatively related to LM332 high expression; therefore, this supports the idea of using LM332 to identify patients where receptor tyrosine kinase inhibitors could be effective.

One of the advantages of this study is the use of multiple discrete datasets to provide triangulation and validation of individual findings. There was generally a strong agreement between the transcript and protein abundance data throughout. However, it should be noted that not all the datasets had available information for all the questions asked. Analysis of survival correlation and drug responsiveness was restricted to the RNA-sequencing data sets and, therefore, would be valuable to confirm these data at the protein level. Each of the datasets naturally contained a different balance of patient age, gender, disease stages and grade, interventions used, and other characteristics. Inevitably, this means that some more subtle effects may have been missed. Indeed, allowing for these differences, the consistency, and strength of the association with LM332 and PAAD, we interpret as being indicative of it being a major contributor to this disease.

## Conclusion

This bioinformatics analysis of the laminin gene family and PAAD showed LM332 genes to be prognostic markers, predicting patient outcomes and drug responsiveness, and assessing their expression can be used to improve pancreatic cancer patient stratification and therapy choices.

## Data Availability

The original contributions presented in the study are included in the article/Supplemental Material; further inquiries can be directed to the corresponding author.

## Supplementary material

SUPPLEMENTARY MATERIAL S1 mRNA and gene-chip data showing upregulation and downregulation of the laminin genes in patients with PAAD. **(A)** Violin plots of the expression levels in laminin transcript data in normal and PAAD patients. **(B)** Violin plots of the expression levels in laminin gene-chip data in normal and PAAD patients. *p* < 0.05 indicates significant changes in TCGA PAAD data.

SUPPLEMENTARY MATERIAL S2 LM332 gene expression levels correlate with one another. **(A)** Gene-chip expression data showing the correlation between LAMA3 with LAMB3, LAMC2, LAMB1, LAMB2, LAMC1, and LAMB3 with LAMC2. **(B)** RNA-seq data comparing all other laminin–laminin gene co-regulation.

SUPPLEMENTARY MATERIAL S3 Protein atlas of laminin in normal and pancreatic cancer specimens. Representative immunohistochemistry images of anti-laminin proteins in normal and pancreatic cancer specimens from Human Protein Atlas (https://www.proteinatlas.org).

SUPPLEMENTARY MATERIAL S4 Association between high expression of LM332 genes and clinicopathological features in PAAD. Chi-squared and Fisher’s exact tests were used to compare the low-expression (first tercile) and high-expression (last tercile) groups for association with clinical features.

SUPPLEMENTARY MATERIAL S5 Correlation between LM332 chains and immune cells. Spearman correlations between immune cells and LAMA3, LAMB3, and LAMC2 genes.

SUPPLEMENTARY MATERIAL S6 Correlation between LM332 chains and immune genes. Spearman correlations between immune-inhibitors, immune-stimulators, MHC molecules, chemokines, chemokine receptors, and LAMA3, LAMB3, and LAMC2 genes.

SUPPLEMENTARY MATERIAL S7 Association between mutations and overall survival. **(A)** Altered LAMA3 gene and overall survival. **(B)** Altered LAMB3 gene and overall survival. **(C)** Altered LAMC2 gene and overall survival.

SUPPLEMENTARY MATERIAL S8 Analysis of genes co-regulated with LM332 in patients with PAAD. Top 50 genes with either positive or negative gene correlation with LAMA3 **(A)**, LAMB3 **(B)**, or LAMC2 **(C)** visualized in heatmaps. For all genes, r2 values >0.7.

SUPPLEMENTARY MATERIAL S9 Protein enrichment and cluster analyses of LM332 genes in patients with PAAD. **(A)** Biological process (BP), cellular component (CC), and molecular function MF enrichment analysis of the top 50 genes most tightly correlated with LAMA3, LAMB3, and/or LAMC2. **(B)** KEGG pathway analysis outcomes from the co-expressed gene list. **(C)** Protein–protein interaction clusters of the genes correlated with LAMA3, LAMB2, and LAMC2.

SUPPLEMENTARY MATERIAL S10 Drug sensitivity of eight hub genes in PAAD in comparison with LM332. Spearman correlation coefficients between the indicated genes and sensitivity to the tested compounds. The size of the circles reflects plotted against the false discovery rates (FDRs), with greater confidence associated with larger circles. Data were generated from the Genomics of Drug Sensitivity in Cancer (GDSC) study, accessed using the GSCA platform.

## Abbreviations

PAAD, pancreatic adenocarcinoma; CPTAC, Clinical Proteomic Tumour Analysis Consortium; ICPC, International Cancer Proteogenome Consortium; TCGA, The Cancer Genome Atlas; HR, hazard ratio; BM, basement membrane.
