# Supplementary figures and images for: Laminin 332 expression levels predict clinical outcomes and chemotherapy response in patients with pancreatic adenocarcinoma
Source: Front Cell Dev Biol. 2023 Sep 15;11:1242706. doi: 10.3389/fcell.2023.1242706 (PMC10540629; doi:10.3389/fcell.2023.1242706)

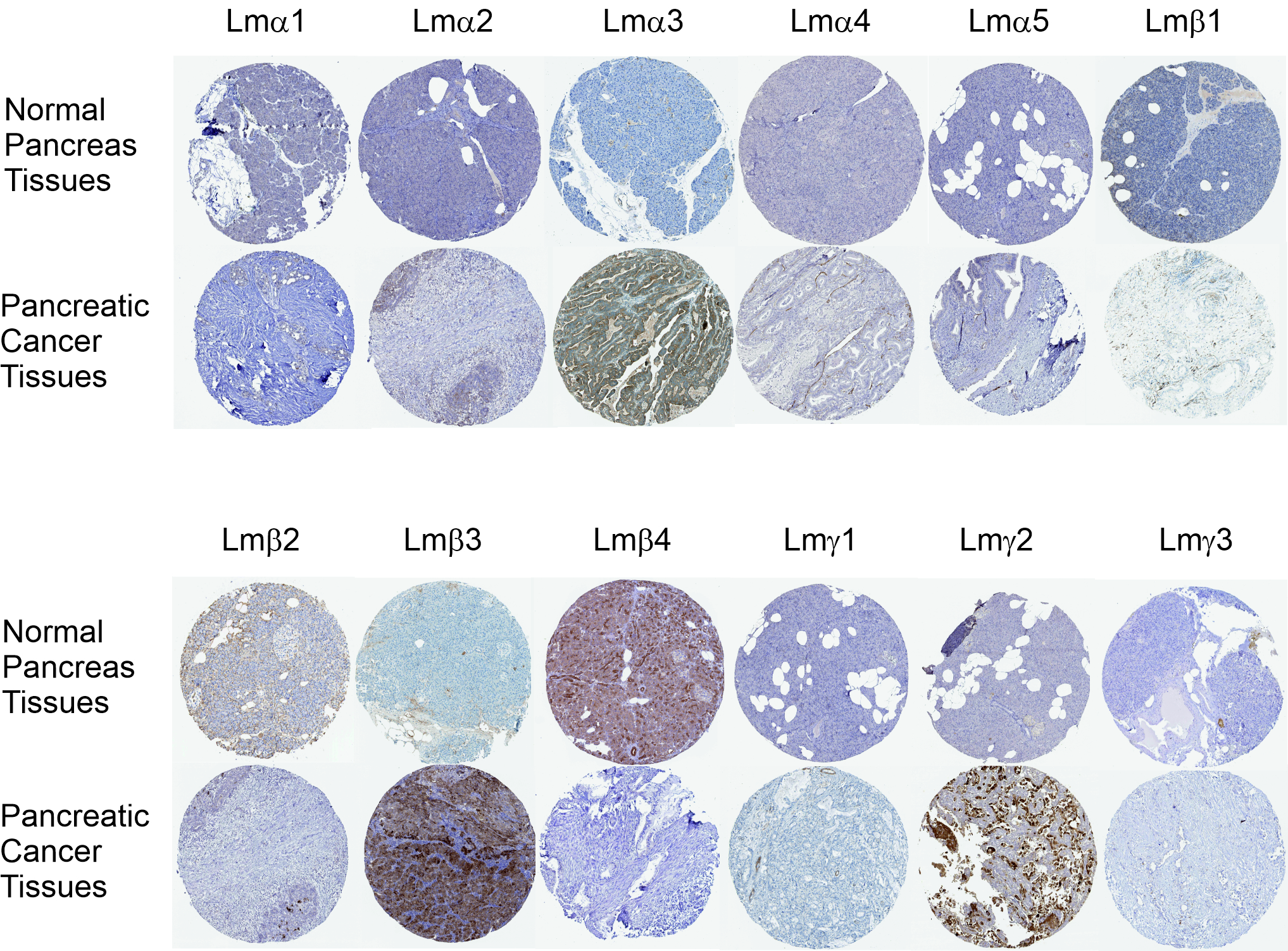

Supplement: Supplementary file 1 [file Image3.JPEG]

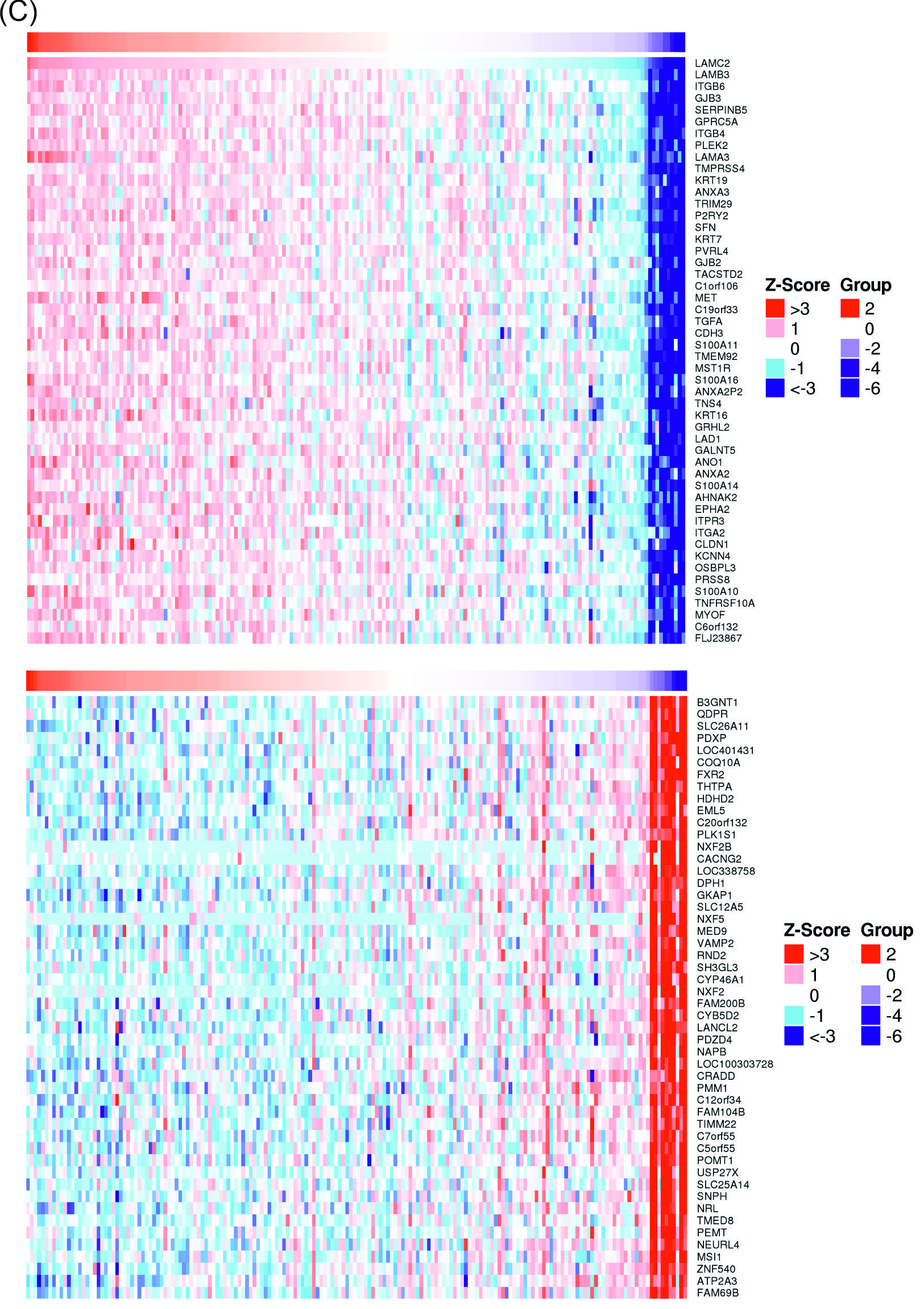

Supplement: Supplementary file 2 [file Image9.JPEG]

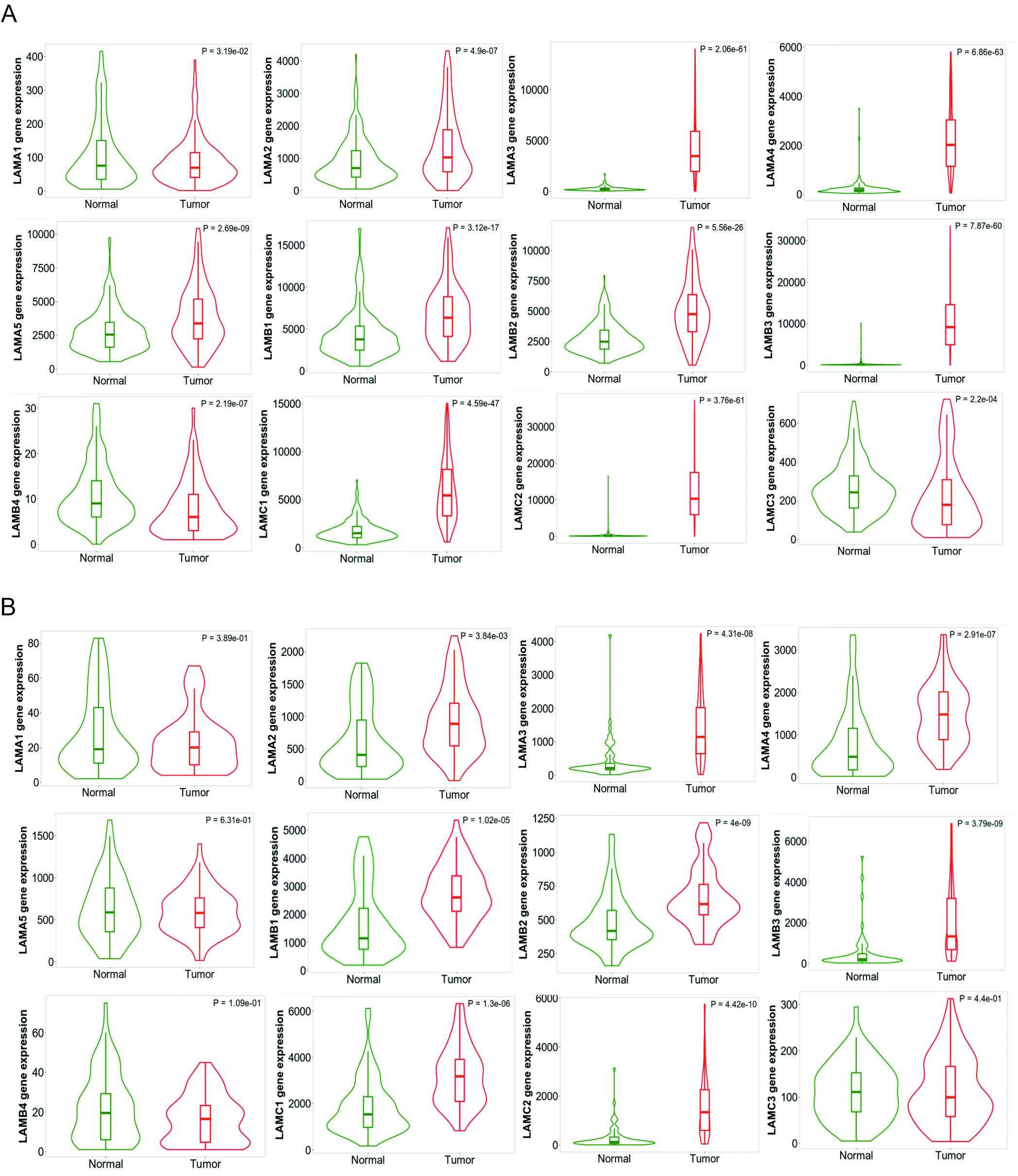

Supplement: Supplementary file 3 [file Image1.JPEG]

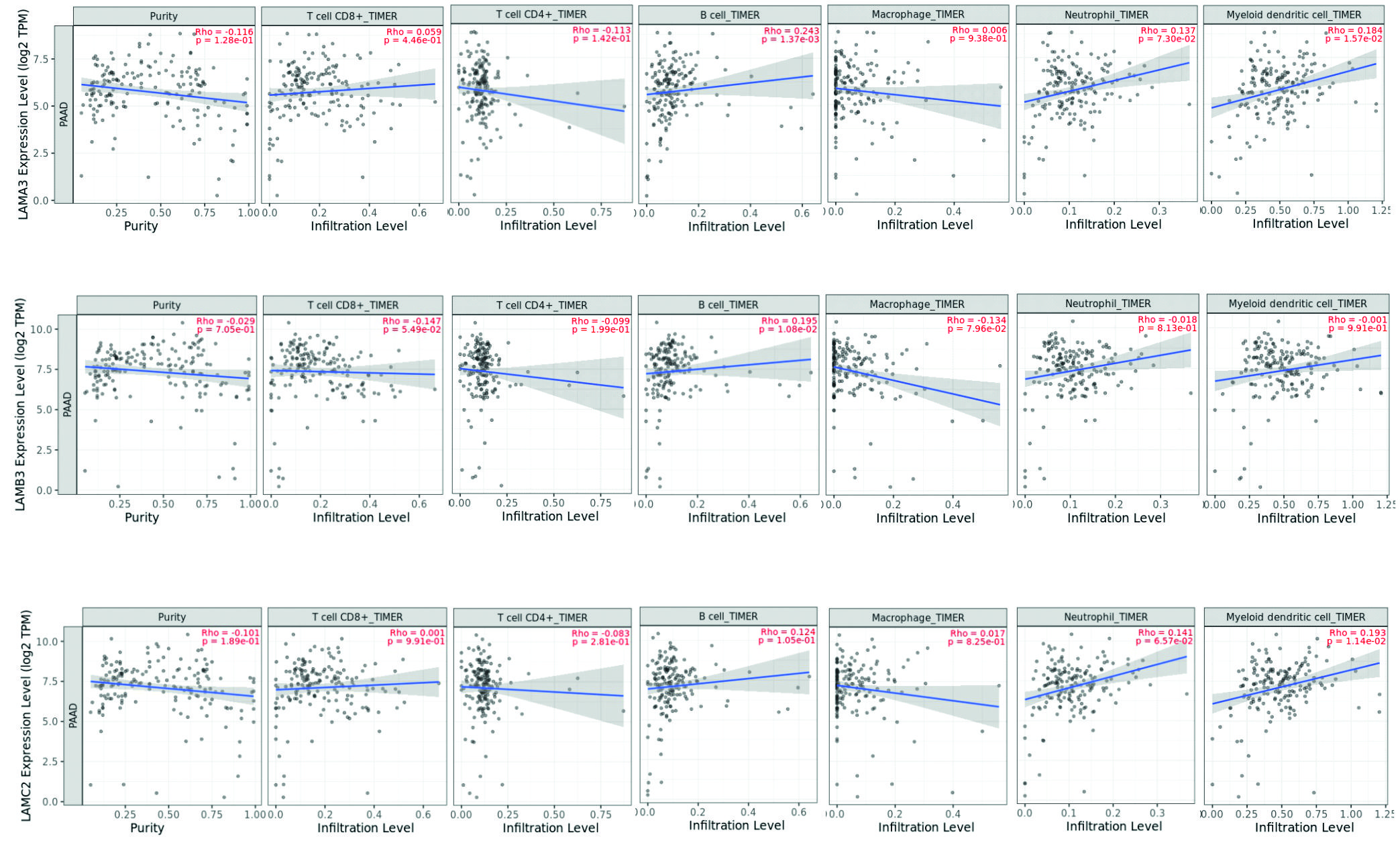

Supplement: Supplementary file 4 [file Image4.JPEG]

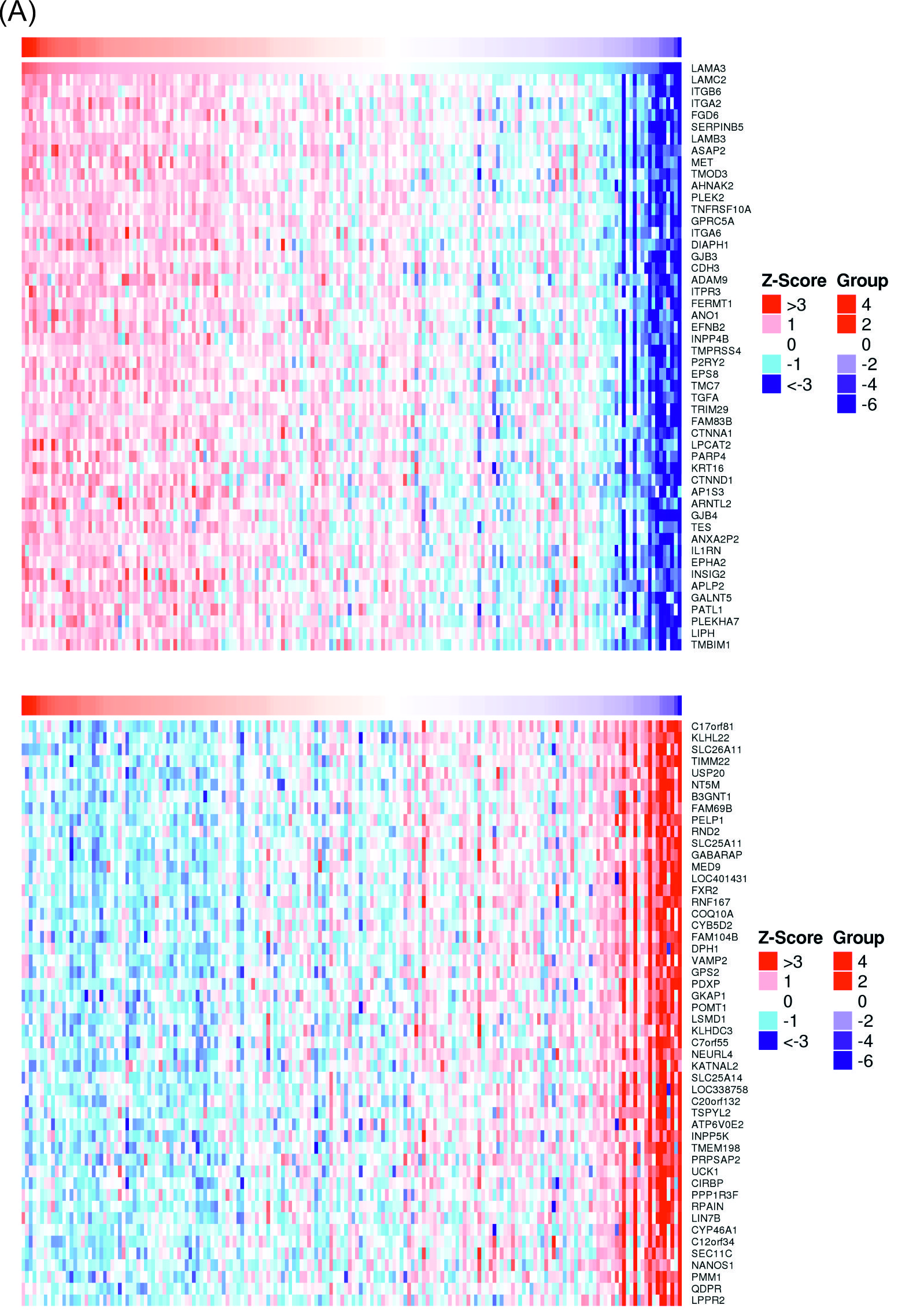

Supplement: Supplementary file 5 [file Image7.JPEG]

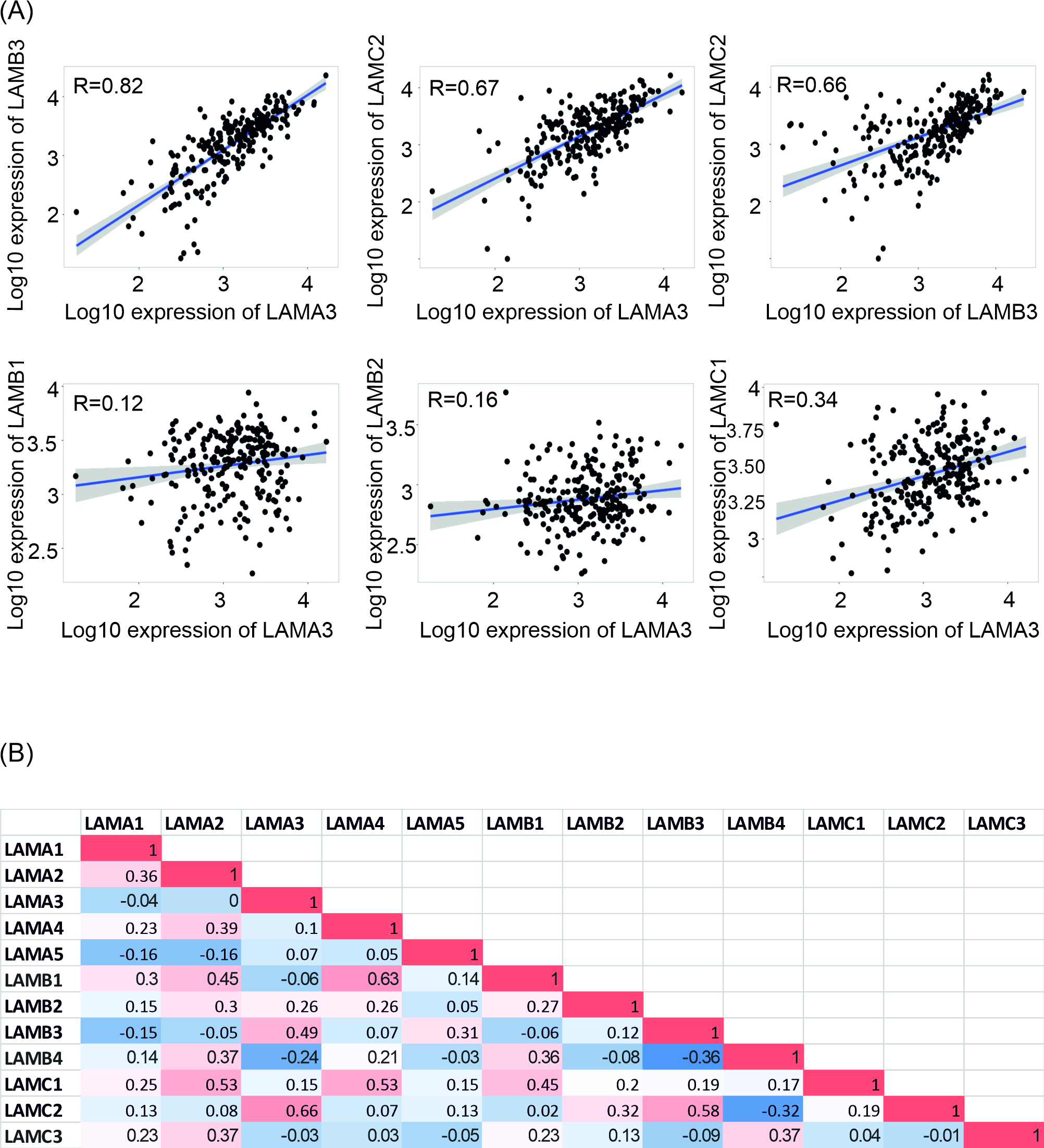

Supplement: Supplementary file 6 [file Image2.JPEG]

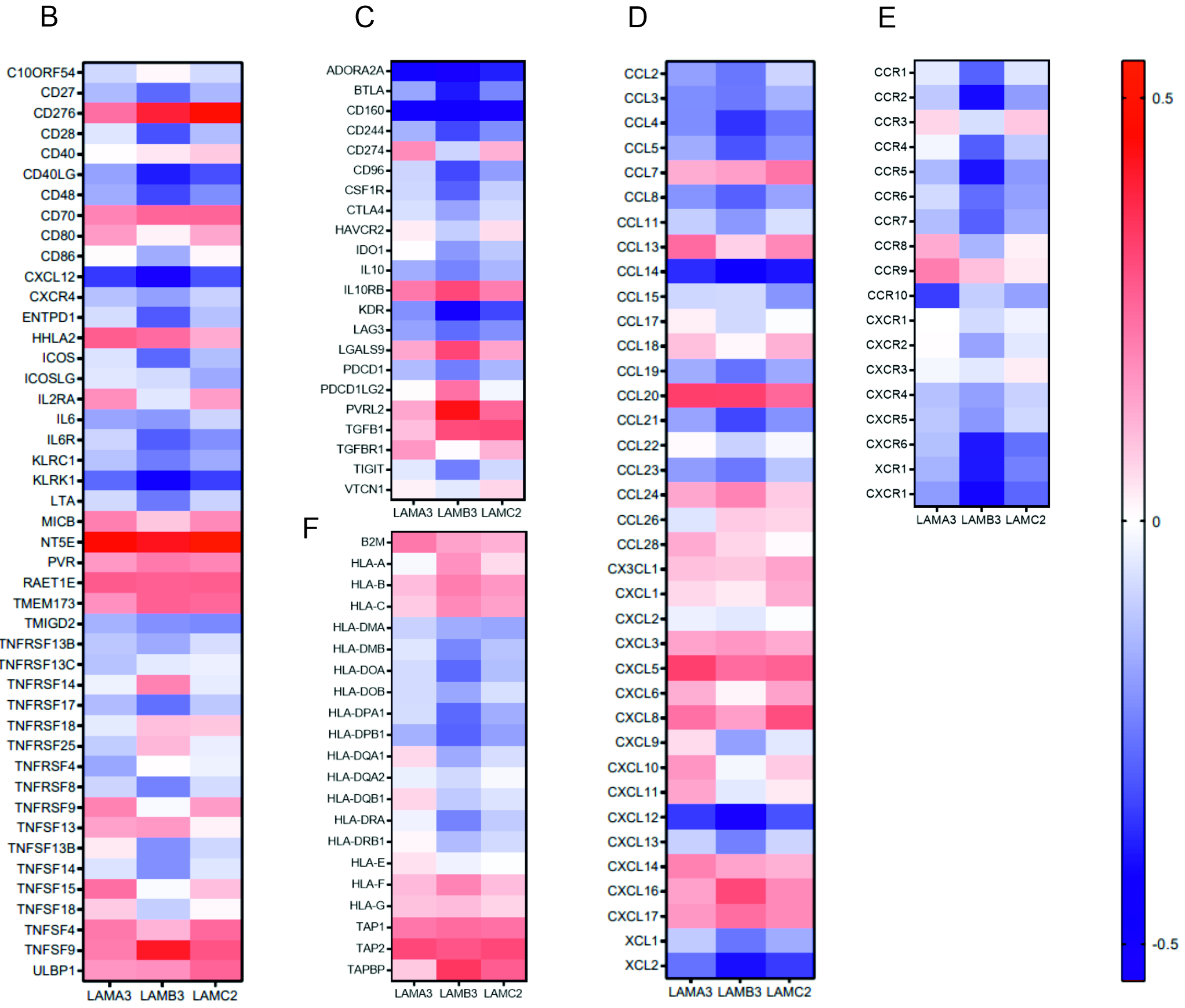

Supplement: Supplementary file 7 [file Image5.JPEG]

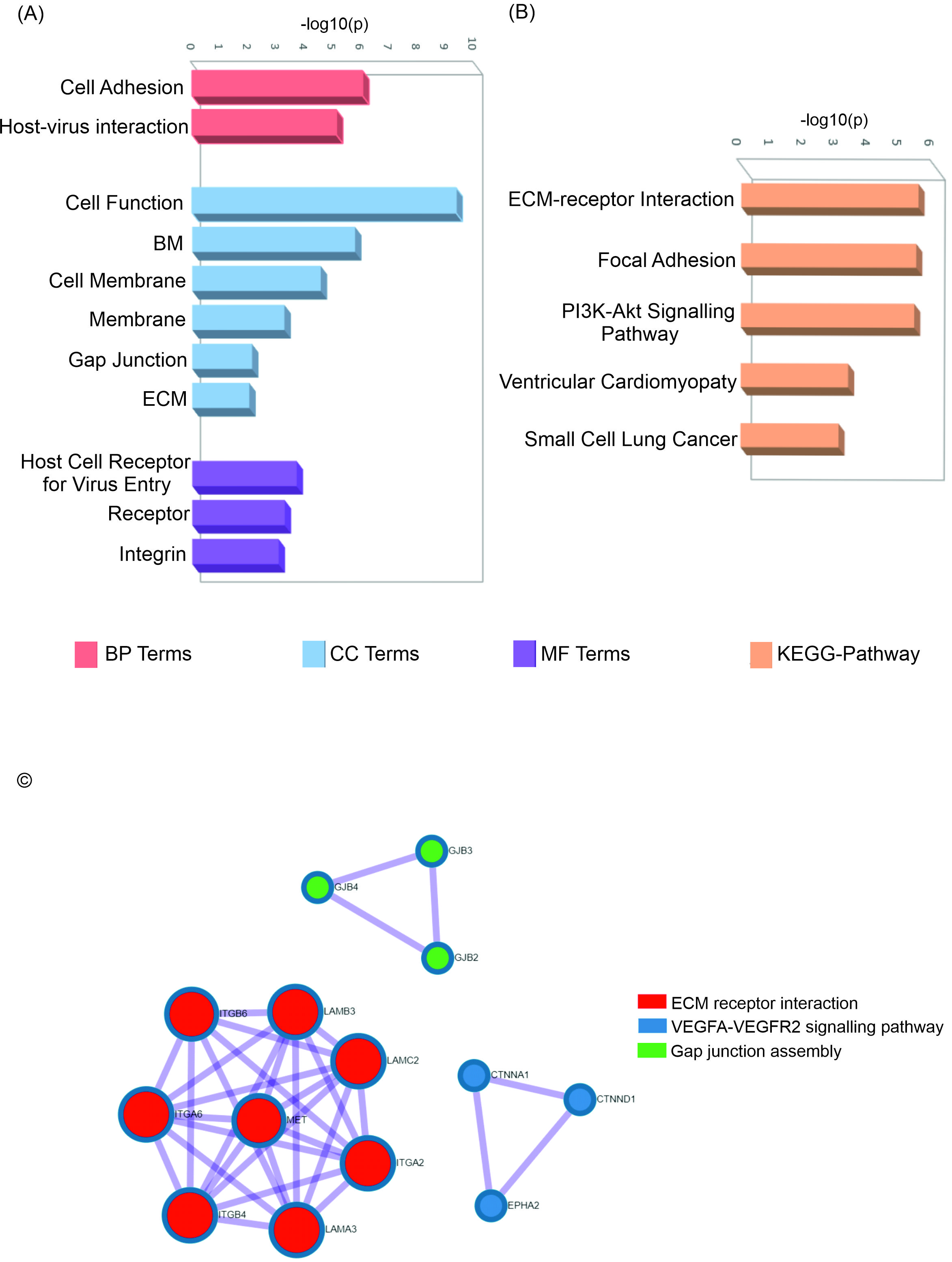

Supplement: Supplementary file 8 [file Image10.JPEG]

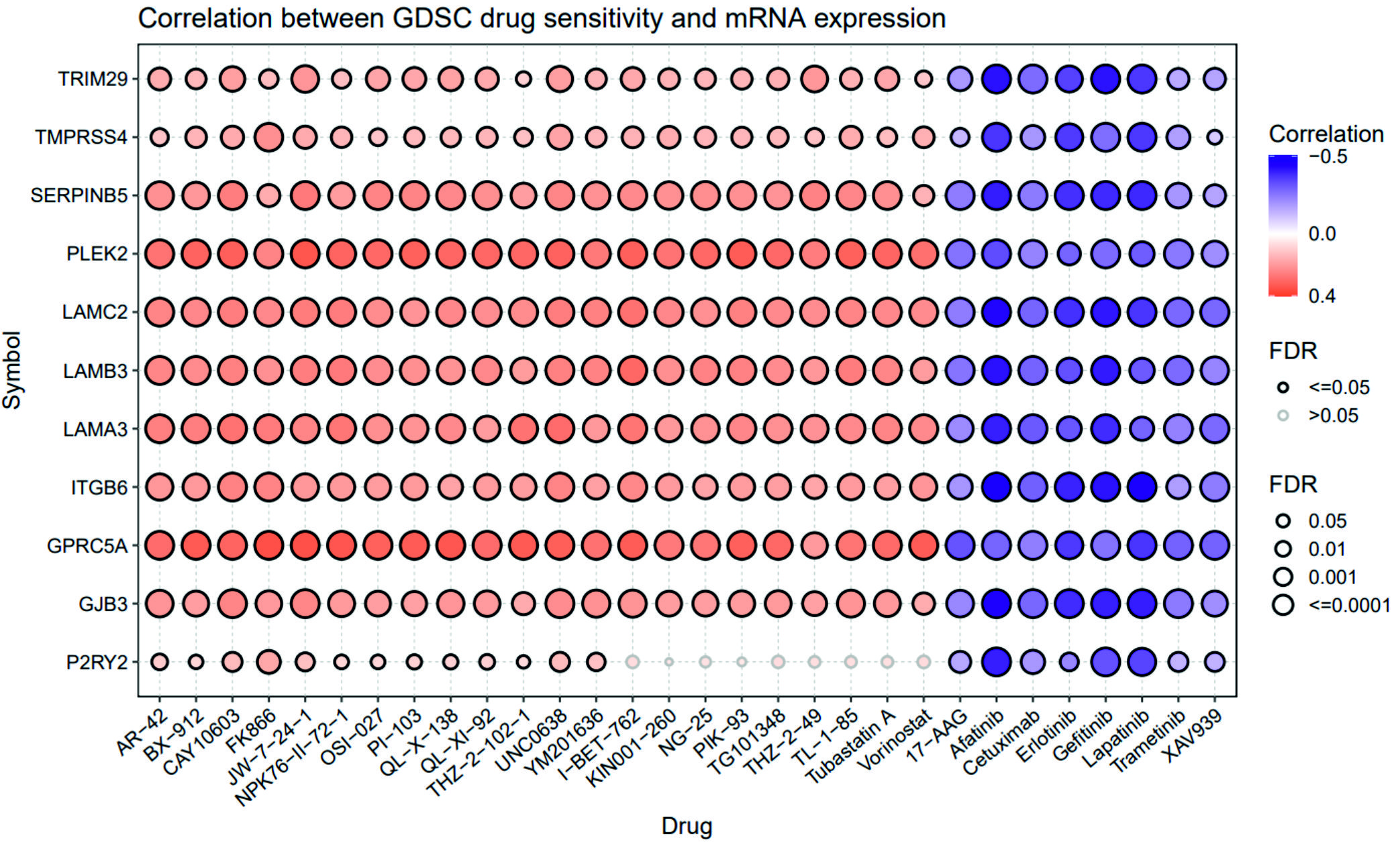

Supplement: Supplementary file 9 [file Image11.JPEG]

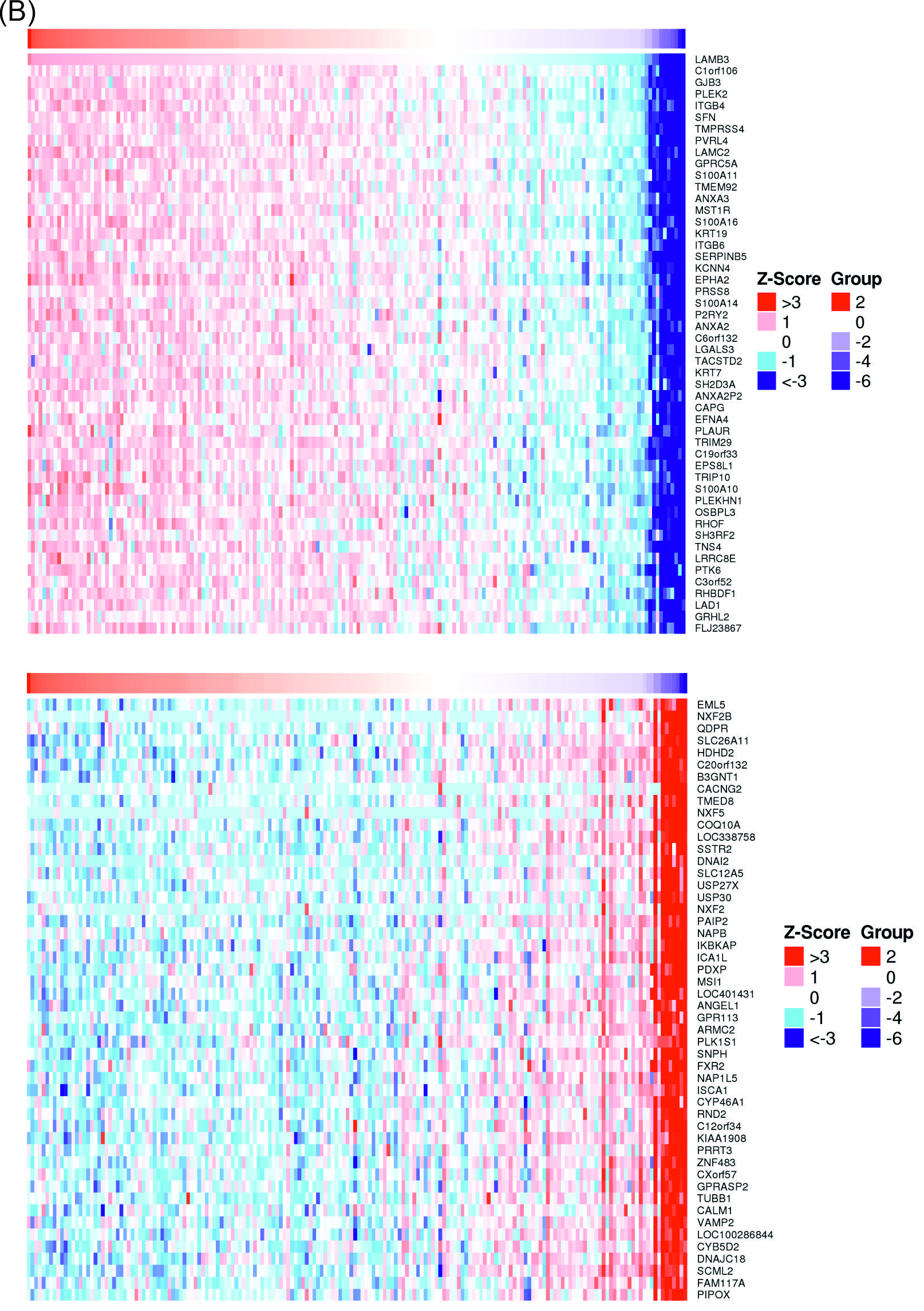

Supplement: Supplementary file 12 [file Image8.JPEG]

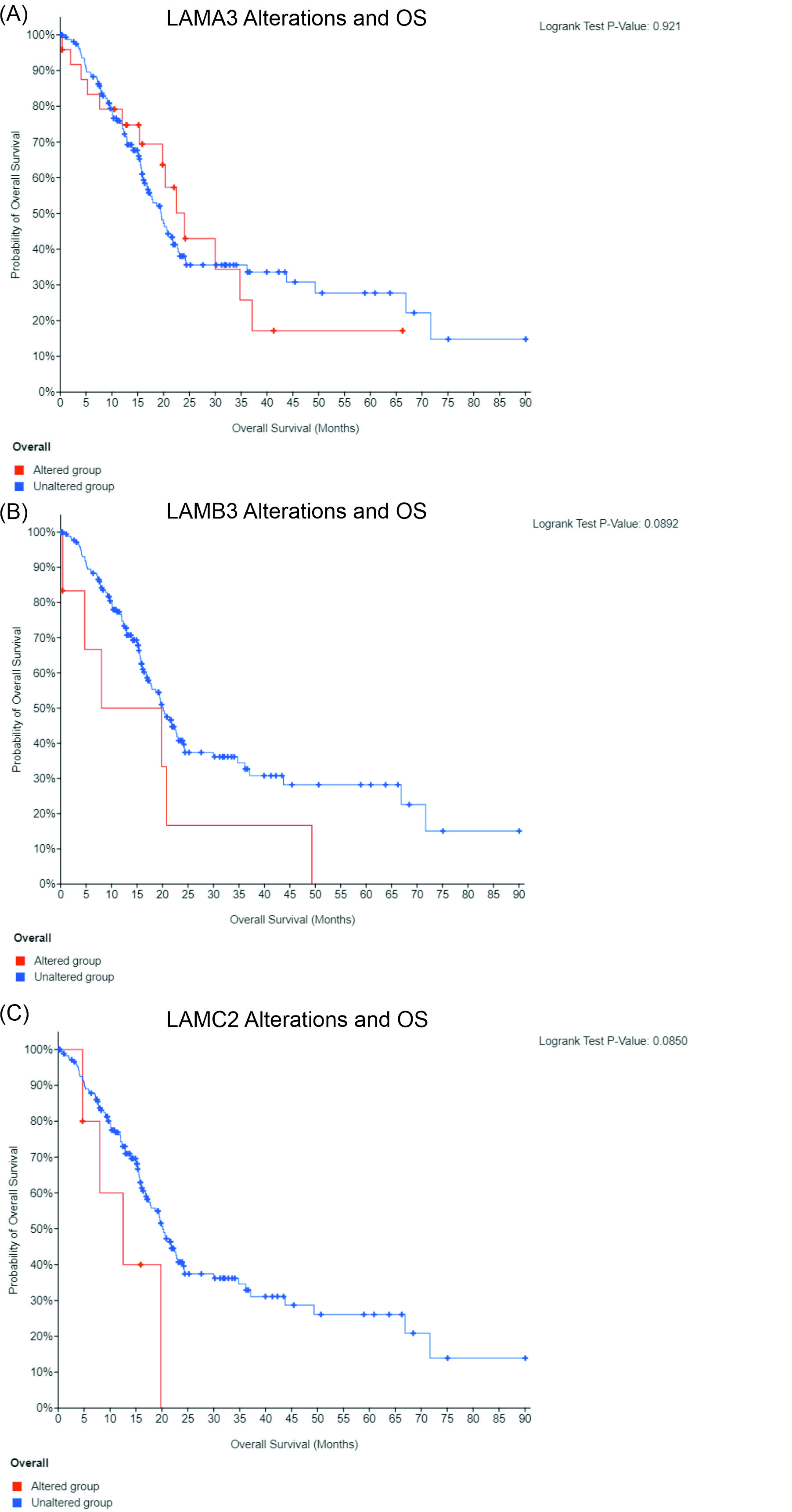

Supplement: Supplementary file 13 [file Image6.JPEG]
