# Supplementary material for: Laminin 332 expression levels predict clinical outcomes and chemotherapy response in patients with pancreatic adenocarcinoma
Source: Front Cell Dev Biol. 2023 Sep 15;11:1242706. doi: 10.3389/fcell.2023.1242706 (PMC10540629; doi:10.3389/fcell.2023.1242706)
Supplement: Supplementary file 10 [file Table2.DOCX]

|  |  | **LAMA3** | | | **LAMB3** | | | **LAMC2** | | |
| --- | --- | --- | --- | --- | --- | --- | --- | --- | --- | --- |
|  | **N/%** | **High**  **N=62** | **Low**  **N=61** | ***p*** | **High**  **N=62** | **Low**  **N=61** | ***p*** | **High**  **N=61** | **Low**  **N=61** | ***p*** |
| **Age** |  |  |  | 0.66 |  |  | 0.66 |  |  | 0.37 |
| <65 | **59 (48)** | 34 (55) | 31 (51) |  | 34 (55) | 31 (51) |  | 33 (54) | 28 (46) |  |
| >=65 | **63 (52)** | 28 (45) | 30 (49) |  | 28 (45) | 30 (49) |  | 28 (46) | 33 (54) |  |
| **Sex** |  |  |  | 0.92 |  |  | **0.02*** |  |  | 0.72 |
| Female | **56 (46)** | 27 (44) | 26 (43) |  | 22 (35) | 34 (56) |  | 27 (44) | 29 (48) |  |
| Male | **66 (54)** | 35 (56) | 35 (57) |  | 40 (65) | 27 (44) |  | 34 (56) | 32 (52) |  |
| **Smoker** |  |  |  | 0.49 |  |  | 0.97 |  |  | 0.92 |
| Yes | **49 (49)** | 27 (54) | 24 (47) |  | 27 (52) | 23 (52) |  | 25 (51) | 24 (50) |  |
| No | **52 (51)** | 23 (46) | 27 (53) |  | 25 (48) | 21 (48) |  | 24 (49) | 24 (50) |  |
| **Alcohol** |  |  |  | 0.88 |  |  | 0.65 |  |  | 1 |
| Yes | **75 (65)** | 36 (61) | 34 (60) |  | 36 (63) | 33 (59) |  | 35 (60) | 35 (60) |  |
| No | **41 (35)** | 23 (39) | 23 (40) |  | 21 (37) | 23 (41) |  | 23 (40) | 23 (40) |  |
| **Diabetes** |  |  |  | 0.23 |  |  | 0.41 |  |  | 0.31 |
| Yes | **31 (32)** | 10 (20) | 15 (30) |  | 12 (23) | 14 (30) |  | 9 (18) | 13 (27) |  |
| No | **67 (68)** | 41 (80) | 35 (70) |  | 40 (77) | 32 (70) |  | 41 (82) | 36 (73) |  |
| **Pancreatitis** |  |  |  | 1 |  |  | **0.03*** |  |  | 0.50 |
| Yes | **10 (10)** | 4 (8) | 3 (6) |  | 8 (16) | 1 (2) |  | 6 (13) | 4 (8) |  |
| No | **86 (90)** | 45 (92) | 45 (94) |  | 42 (84) | 45 (98) |  | 42 (87) | 44 (92) |  |
| **Family history of cancer** | | |  | 0.65 |  |  | 0.10 |  |  | 0.91 |
| Yes | **47 (59)** | 21 (54) | 23 (59) |  | 14 (39) | 21 (58) |  | 21 (53) | 21 (54) |  |
| No | **32 (41)** | 18 (46) | 16 (41) |  | 22 (61) | 15 (42) |  | 19 (47) | 18 (46) |  |
| **Anatomic subdivision** | | |  | 0.64 |  |  | 0.71 |  |  | 0.54 |
| Head of Pancreas | **92 (75)** | 47 (76) | 44 (72) |  | 46 (74) | 47 (77) |  | 46 (75) | 43 (70) |  |
| Other | **30 (25)** | 15 (24( | 17 (28) |  | 16 (26) | 14 (23) |  | 15 (25) | 18 (30) |  |
| **Radiation therapy** | |  |  | 0.62 |  |  | 0.79 |  |  | 0.49 |
| Yes | **28 (25)** | 15 (26) | 18 (31) |  | 16 (28) | 17 (30) |  | 14 (25) | 17 (30) |  |
| No | **84 (75)** | 42 (74) | 41 (69) |  | 42 (72) | 40 (70) |  | 43 (75) | 39 (70) |  |
| **Residual tumour** | | |  | **0.001*** |  |  | 0.18 |  |  | **0.01*** |
| R0 | **76 (66)** | 31 (53) | 41 (77) |  | 35 (64) | 43 (76) |  | 31 (56) | 43 (80) |  |
| R1 | **35 (30)** | 27 (47) | 9 (17) |  | 19 (34) | 11 (19) |  | 24 (44) | 11 (20) |  |
| R2 | **4 (4)** | 0 (0) | 3 (6) |  | 1 (2) | 3 (5) |  | - | - |  |
| **Histologic Grade** | |  |  | **0.01*** |  |  | **0.01*** |  |  | **0.01*** |
| G1 | **25 (21)** | 5 (8) | 18 (30) |  | 4 (6) | 18 (30) |  | 6 (10) | 18 (31) |  |
| G2 | **64 (53)** | 35 (56) | 25 (42) |  | 37 (60) | 28 (47) |  | 33 (54) | 28 (47) |  |
| G3 | **31 (25)** | 22 (36) | 15 (25) |  | 21 (34) | 13 (21) |  | 22 (36) | 11 (19) |  |
| G4 | **1 (1)** | 0 (0) | 2 (3) |  | 0 (0) | 1 (2) |  | 0 (0) | 2 (3) |  |
| **Pathologic Stage** | |  |  | **0.04*** |  |  | 0.10 |  |  | 0.07 |
| Stage I | **16 (13)** | 2 (3) | 10 (17) |  | 4 (7) | 12 (20) |  | 4 (7) | 13 (22) |  |
| Stage II | **96 (80)** | 55 (89) | 47 (79) |  | 54 (87) | 45 (76) |  | 52 (85) | 43 (73) |  |
| Stage III | **4 (3)** | 2 (3) | 1 (2) |  | 2 (3) | 1 (2) |  | 2 (3) | 1 (2) |  |
| Stage IV | **5 (4)** | 3 (5) | 1 (2) |  | 2 (3) | 1 (2) |  | 3 (5) | 2 (3) |  |
| **T stage** |  |  |  | 0.15 |  |  | 0.32 |  |  | 0.14 |
| T1 | **5 (4)** | 0 (0) | 3 (5) |  | 2 (3) | 4 (7) |  | 1 (2) | 3 (5) |  |
| T2 | **17 (14)** | 6 (9) | 10 (17) |  | 6 (10) | 11 (18) |  | 5 (8) | 12 (20) |  |
| T3 | **96 (80)** | 55 (89) | 45 (76) |  | 53 (85) | 43 (73) |  | 54 (88) | 43 (73) |  |
| T4 | 3 (2) | 1 (2) | 1 (2) |  | 1 (2) | 1 (2) |  | 1 (2) | 1 (2) |  |
| **N stage** |  |  |  | 0.18 |  |  | 0.91 |  |  | 0.29 |
| N0 | 33 (28) | 12 (20) | 17 (30) |  | 19 (31) | 18 (32) |  | 16 (27) | 20 (36) |  |
| N1 | 84 (72) | 49 (80) | 39 (70) |  | 42 (69) | 38 (68) |  | 44 (73) | 36 (64) |  |
